# Supplementary material for: Fixation and Spread of Somatic Mutations in Adult Human Colonic Epithelium
Source: Cell Stem Cell. 2018 Jun 1;22(6):909–918.e8. doi: 10.1016/j.stem.2018.04.020 (PMC5989058; doi:10.1016/j.stem.2018.04.020)
Supplement: Document S1. Figures S1–S4 and Tables S1–S3 [file mmc1.pdf]

Cell Stem Cell, Volume *22*

Supplemental Information

Fixation and Spread of Somatic Mutations in Adult Human Colonic Epithelium

Anna M. Nicholson, Cora Olpe, Alice Hoyle, Ann-Sofie Thorsen, Teja Rus, Mathilde Colombé, Roxanne Brunton-Sim, Richard Kemp, Kate Marks, Phil Quirke, Shalini Malhotra, Rogier ten Hoopen, Ashraf Ibrahim, Cecilia Lindskog, Meagan B. Myers, Barbara Parsons, Simon Tavaré, Mark Wilkinson, Edward Morrissey, and Douglas J. Winton

Figure S1. Image analysis pipelines. Related to Figure 1 and STAR methods

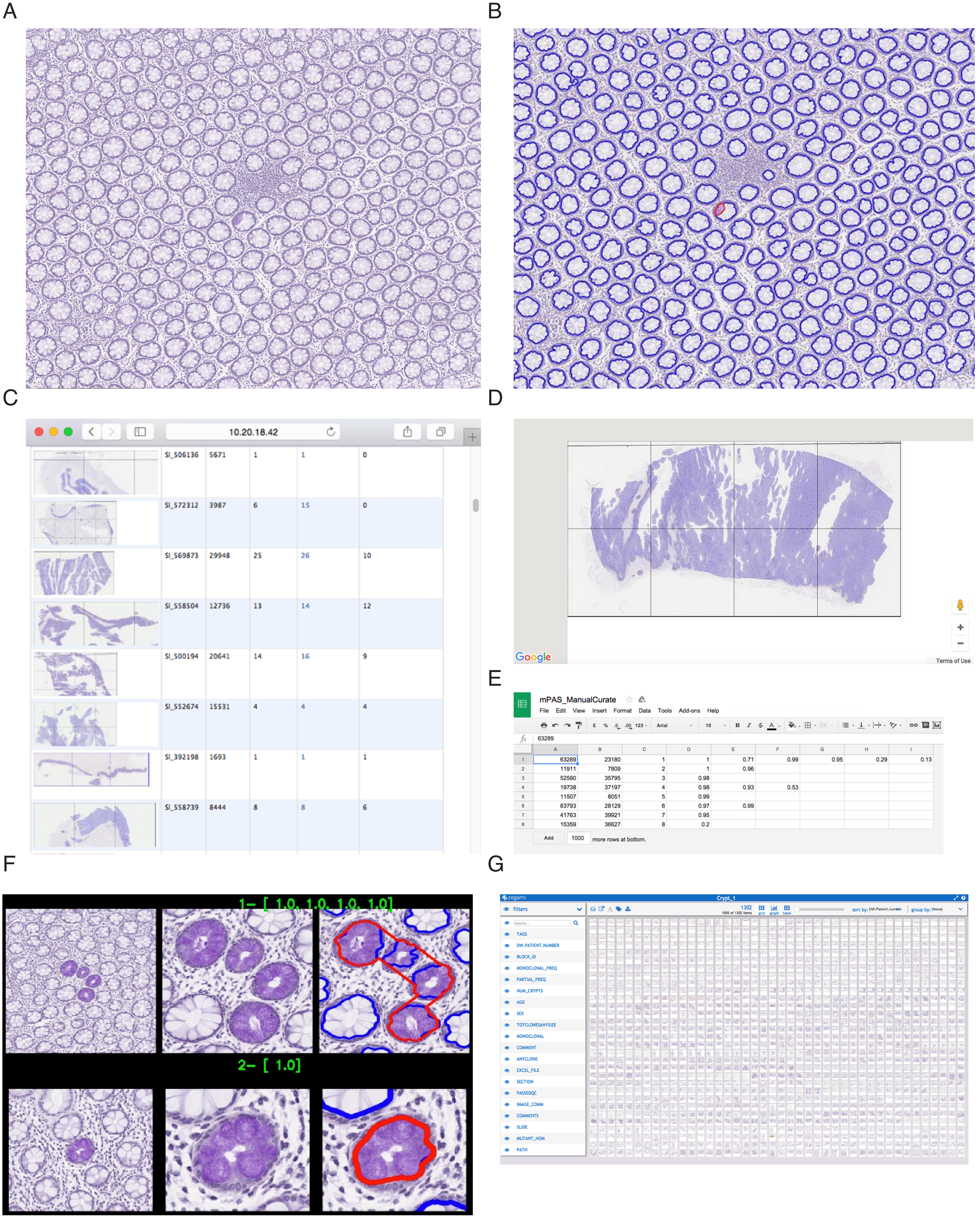

Figure S2. Determining a de novo mutation rate in human colon.
Related to Figure 1 and STAR methods

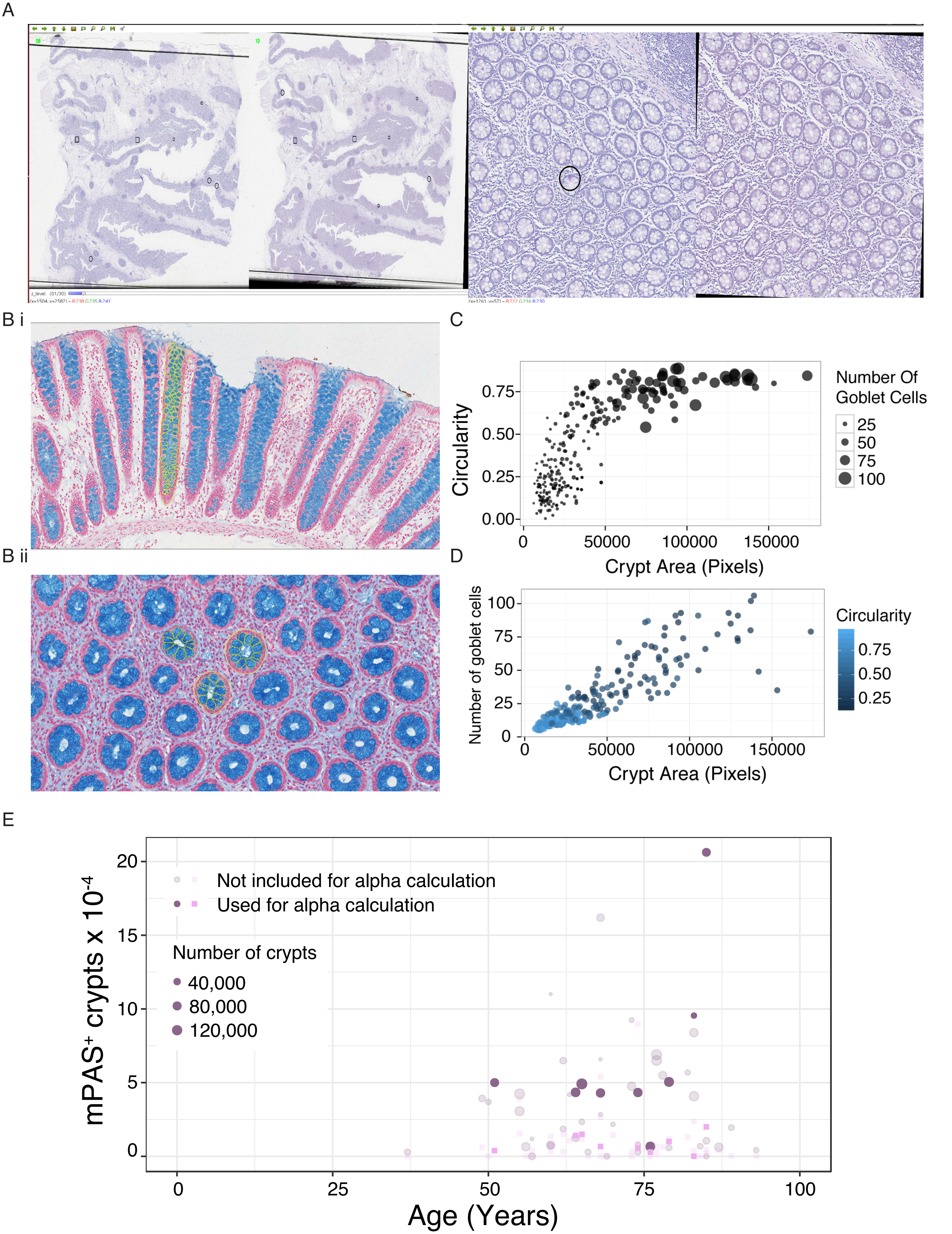

Figure S3. Split MAOA data set, conversion rates and sequential mutation simulations, Related to Figure 2, Figure 3 and STAR Methods

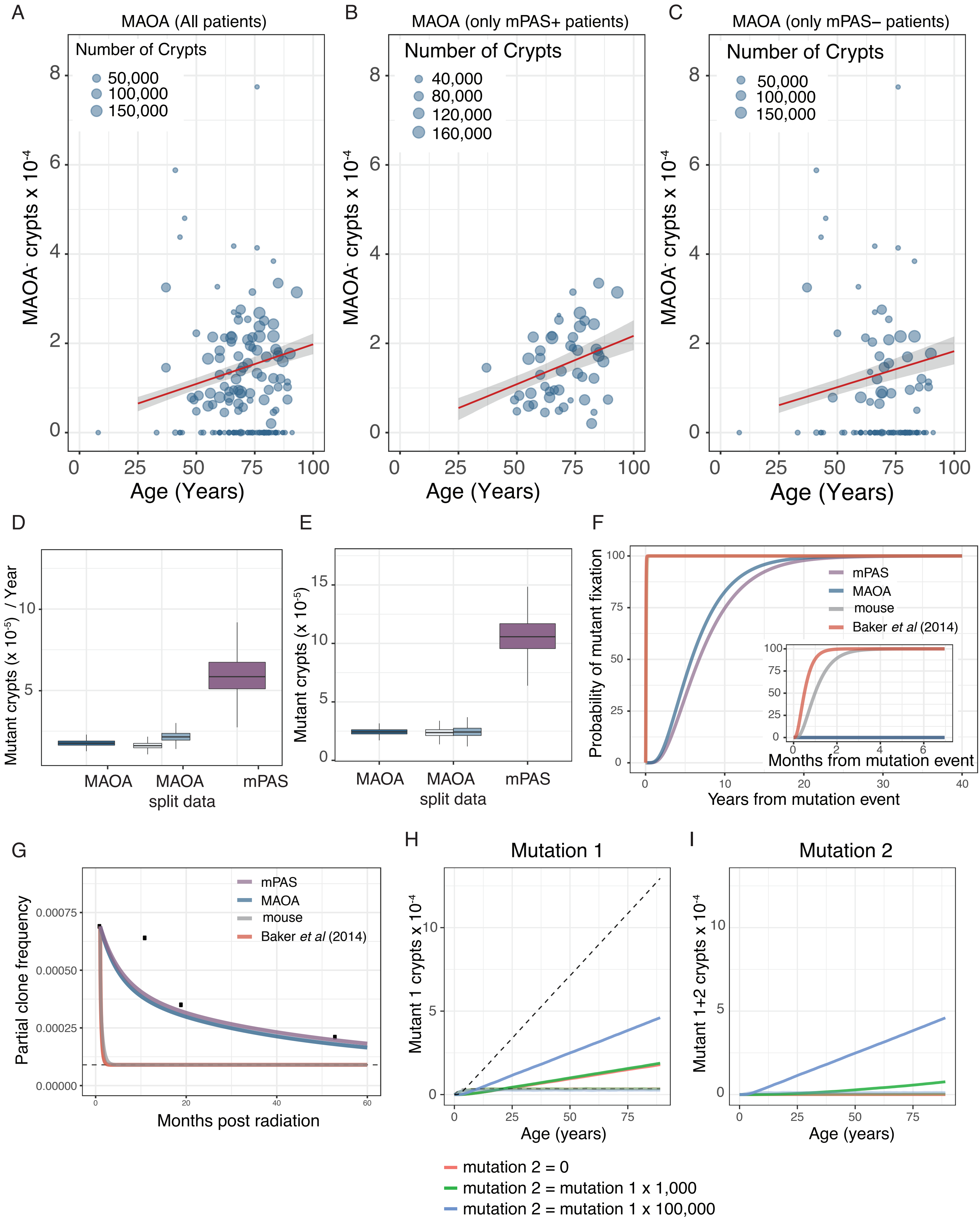

Figure S4. Effects of fission and fusion on patch size in a WT and oncogenic setting, Related to Figure 4 and STAR Methods.

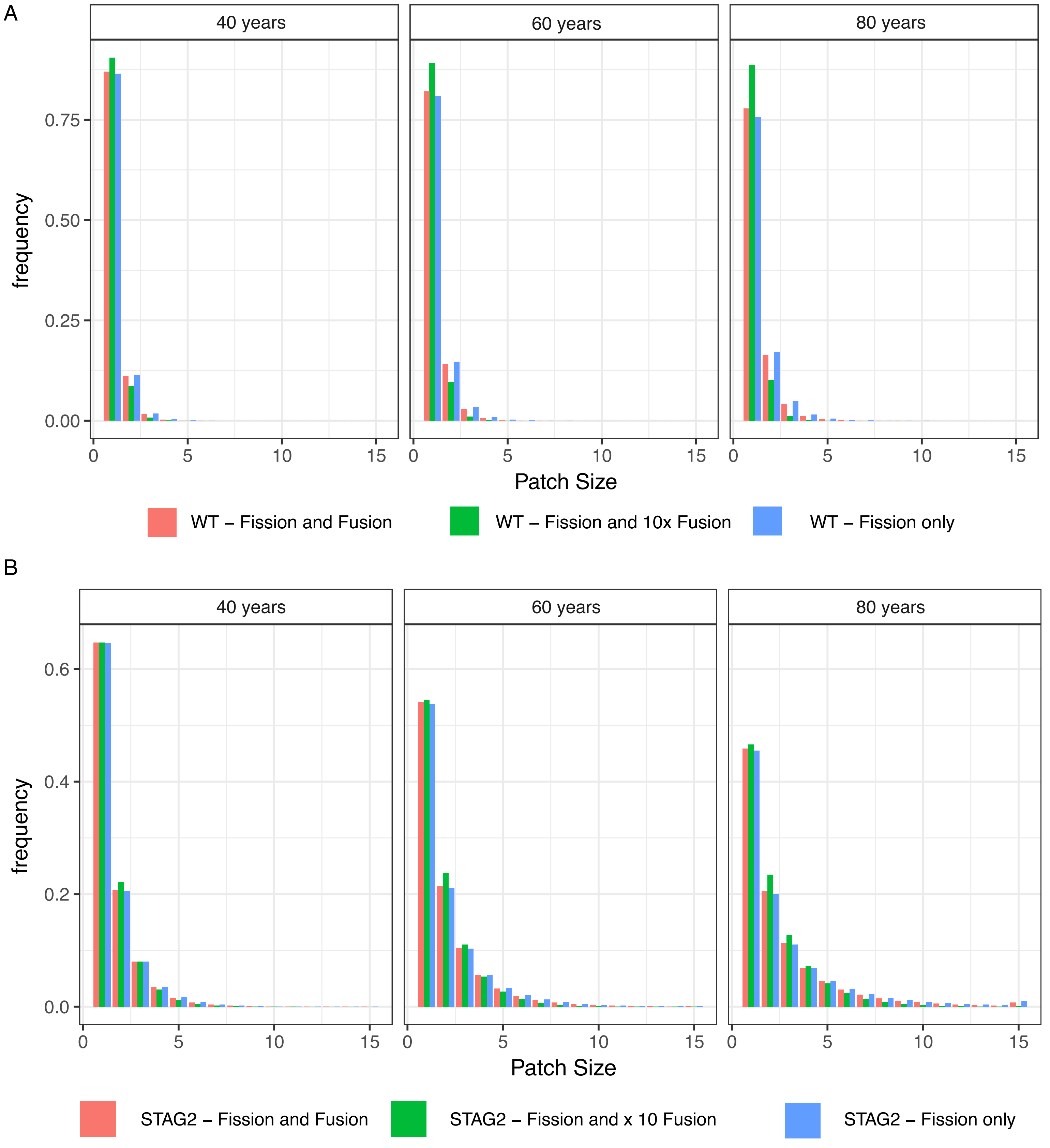

Supplemental Figure Legends

Figure S1. Image analysis pipelines. Related to Figure 1 and STAR Methods

(A, B) mPAS stained sections are scanned and images processed to identify crypts and candidate clones within the section. Before processing (A). After processing (B) crypts are outlined (blue) and mPAS positive clones identified (a PPC in this case) (red) (C) View of web-browser populated with tiles for each mPAS stained section, a linked image ID, calculated crypt number and number of detected clones. (D) An example of the use of Google maps to visualise images. Each processed mPAS stained section can be opened using Google maps via the link in the web-browser to visualise detected clones within the tissue section. (E) A Google sheet is produced for each stained and processed section with information on all clones detected, including co-ordinates and number of crypts within a patch. Using this information each clone is manually validated. (F) An image list of candidate clones are viewed from a link in the web-browser, each clone is numbered and a low power and high power pre-processed image of the clone is shown alongside the processed image. Within the list, images are ordered by stain intensity and size. (G) View of the Zegami software, Zegami was used to aid in visualisation of the many hundreds of slides included in this study. Alongside the images, complete data including patient information such as age and sex as well as number of clones can be observed within the software.

Figure S2. Determining a de novo mutation rate in human colon. Related to Figure 1 and STAR Methods

(A) Rotated serial sections can be viewed side by side on the screen. The detected clones are highlighted on each section, and a slider seen on the bottom of the screen enables movement through the serial sections. Clicking on an area of the low power section brings up a high power image of that area on both serial sections in view, this enables the same crypt to be visualised throughout the block. (B) Sections from human colon stained using Alcian blue/Periodic Acid Schiff's. Images from over 200 crypts were annotated using Fiji (ImageJ) to determine the crypt area and the number of goblet cells within each crypt scored (yellow lines). Crypts from both *en edge* (i) and *en face* (ii) sections were included in the analysis. (C) Dot plot shows roundness (0-1) against crypt area in pixels, where the size of the dot is relative to the number of goblet cells within the crypt. (D) Dot plot shows the number of goblet cells against crypt area in pixels, where the more circular the crypt the darker blue the point. Data from manual scoring shows larger crypts contain more goblet cells, and that rounder crypts are generally smaller. (E) For each patient the frequencies of $mPAS^+$ WPC (circles) and PPC (squares) are plotted against patient age (years), in this plot the individuals used to calculate the mutation rate are highlighted in darker points compared to those not used for the calculation.

Figure S3. Split MAOA data set, conversion rates and sequential mutation simulations. Related to Figure 2, Figure 3 and STAR Methods

(A-C) Frequency plots of WPC for MAOA deficient clones with regression analyses showing$D\Delta C_{fix}$ for MAOA plotted in red with 95% CI shaded in grey. All 152 patients are included in (A), while the 48 mPAS+ patients are only plotted in (B) and the 104 mPAS- patients are plotted in (C). (D) Box plot showing the ΔC_{fix} found for

each patient set including the credible intervals, and (E) box plot showing the fitted frequency of partially populated crypts along with their credible intervals in each patient set. (F-G) Comparison of monoclonal conversion rates. Mutant fixation times by crypt monoclonal conversion plotted using parameters derived from: (F) spontaneous mPAS and MAOA clone data and those stated in Baker *et al* (2014) and for mouse colonic epithelium (Vermeulen *et al*, (2013)). (G) Model predictions of monoclonal conversion times using published human "pulse chase" data for mPAS PPC clones following irradiation. Points are data from Campbell *et al* 1996. Lines show model inference predictions. (H-I) Results from simulations of sequential mutations. The graphs show the full and partial clone frequency for simulations of double mutations. (G) The dotted lines show the observed data for STAG2. The coloured lines show the predicted clone frequencies for a comparable gene (Mutation 1) assuming it is neutral with respect to stem cell replacement but conferring an altered mutation probability at the rates shown on a second locus (Mutation 2) that confers an advantage on replacement. (I) Shows the predicted frequencies of clones containing both Mutation 1 and Mutation 2.

Figure S4. Effects of fission and fusion on patch size in a WT and oncogenic setting. Related to Figure 4 and STAR Methods

Results of simulations for different fusion rates showing that relative patch size is dominated by the fission rate, with the fusion rate having to be 10 times higher than fission to see an appreciable effect. (A) Using mPAS inferred parameters and varying the fusion rate. The fusion rates used are 0, balanced fusion and fission rates and fusion rate x 10. (B) Using Stag2 inferred parameters with the fusion rates as described for A.

Table S1. Summary of patients included in the study, Related to STAR Methods

**Characteristic**		**Set (n = 187)**
Age	Minimum	8
	Maximum	93
	Median	69
Sex	Male	97
	Female	89
Colonic Region	Left	100
	Right	66
	Unknown	21

Table S2 – Summary of X-chromosome encoded proteins investigated as part of this study. Related to STAR Methods

Antigen	Antibody	Conc.	Crypts screened	Comments	DNA repair/pro-oncogenic associated
Apex2	HPA030872	0.5ug/ml	73,529	No clones detected	Yes
CXorf57	HPA001374	0.5ug/ml	125,150	No clones detected	No
GPKOW	HPA001894	4ug/ml	83,567	No clones detected	No
MAOA	SC-271123<br>HPA059299	1ug/ml<br>1ng/ul	6,815,804	1,052 clones detected<br>Validation	No
POLA1	HPA002947	0.1mg/ml	168,395	No clones detected	Yes
RbAp48/RbAp46	R3779	1.2ug/ml	121,377	No clones detected	Yes
Stag2	LS-B11284<br>HPA002857	1ug/ml<br>2.1ug/ml	5,402,413	8,743 clones detected<br>Validation	Yes
THOC2	HPA047921	0.1mg/ml	178,854	No clones detected	No

Table S3 – Data from normal colonic mucosa of 20 patients showing *KRAS*G12D mutant allele frequency (Log10). Related to Figure 4 and STAR Methods

Sample	Age	Log10 *KRAS* codon 12 G12D mutant frequency (GAT)
1	30	-4.61
2	31	-4.54
3	33	-4.58
4	34	-4.68
5	46	-4.19
6	48	-5.14
7	49	-4.87
8	49	-5.19
9	50	-3.78
10	50	-4.57
11	52	-4.57
12	53	-4.59
13	54	-3.32
14	56	-2.83
15	58	-3.94
16	72	-3.82
17	79	-4.72
18	79	-3.85
19	88	-3.25
20	94	-4.47
