# Supplementary material for: Fixation and Spread of Somatic Mutations in Adult Human Colonic Epithelium
Source: Cell Stem Cell. 2018 Jun 1;22(6):909–918.e8. doi: 10.1016/j.stem.2018.04.020 (PMC5989058; doi:10.1016/j.stem.2018.04.020)
Supplement: Document S2. Article plus Supplemental Information [file mmc2.pdf]

Short Article

Cell Stem Cell

Fixation and Spread of Somatic Mutations in Adult Human Colonic Epithelium

Graphical Abstract

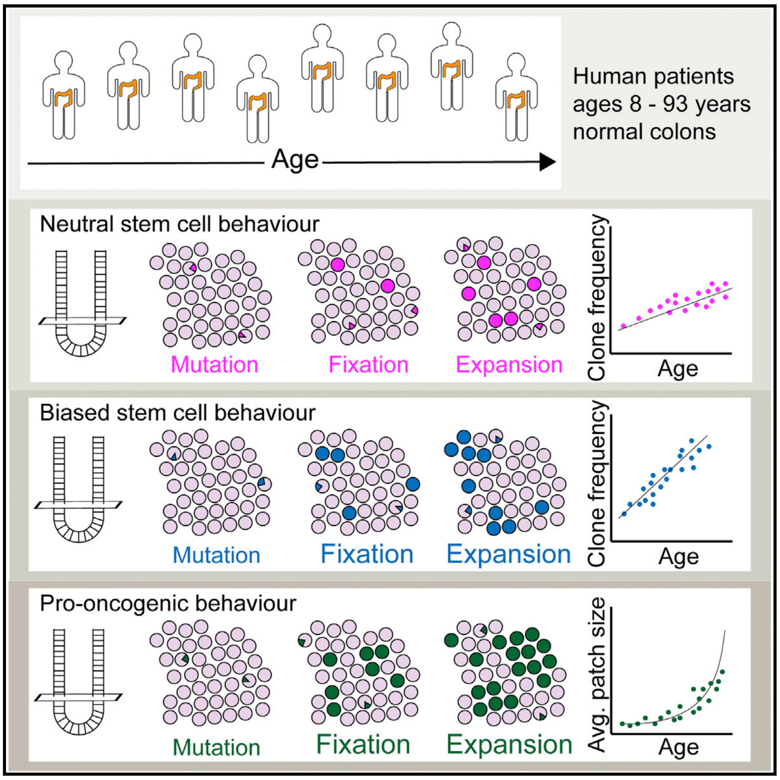

Highlights

- Colonic stem cell dynamics predict lifetime mutant allele frequencies
- Mutant clone fixation in colonic crypts takes years due to slow stem cell turnover
- Crypt fission enables lateral expansion of mutant clones
- Biases in both fixation and expansion increase age-related pro-oncogenic burden

Authors

Anna M. Nicholson, Cora Olpe, Alice Hoyle, ..., Mark Wilkinson, Edward Morrissey, Douglas J. Winton

Correspondence

edward.morrissey@imm.ox.ac.uk (E.M.), doug.winton@cruk.cam.ac.uk (D.J.W.)

In Brief

Winton and colleagues describe stem cell dynamics in normal human colon to identify the efficiency of clone fixation within the epithelium and the rate of subsequent lateral expansion. Against these benchmarks biased stem cell behaviors advantaged in both fixation and expansion can be quantified to predict the age-related burden of pro-oncogenic mutation.

Nicholson et al., 2018, Cell Stem Cell 22, 909–918
June 1, 2018 © 2018 The Authors. Published by Elsevier Inc.
https://doi.org/10.1016/j.stem.2018.04.020

CellPress

Cell Stem Cell
Short Article

Fixation and Spread of Somatic Mutations in Adult Human Colonic Epithelium

Anna M. Nicholson,[1] Cora Olpe,[1,2] Alice Hoyle,[1] Ann-Sofie Thorsen,[1] Teja Rus,[1] Mathilde Colombé,[1] Roxanne Brunton-Sim,[3] Richard Kemp,[1] Kate Marks,[4] Phil Quirke,[4] Shalini Malhotra,[5] Rogier ten Hoopen,[5] Ashraf Ibrahim,[5] Cecilia Lindskog,[6] Meagan B. Myers,[7] Barbara Parsons,[7] Simon Tavaré,[1] Mark Wilkinson,[3] Edward Morrissey,[8,*] and Douglas J. Winton[1,9,*]

[1]Cancer Research-UK Cambridge Institute, Li Ka Shing Centre, Robinson Way, Cambridge CB2 0RE, UK
[2]Wellcome Trust-Medical Research Council, Cambridge Stem Cell Institute, Cambridge, UK
[3]Norwich Research Park BioRepository, James Watson Road, Norwich NR4 7UQ, UK
[4]Pathology and Tumour Biology, Level 4, Wellcome Trust Brenner Building, St. James University Hospital, Beckett Street, Leeds LS9 7TF, UK
[5]Department of Histopathology, Box 235, CUHFT, Cambridge, UK
[6]Department of Immunology, Genetics and Pathology, Science for Life Laboratory, Rudbeck Laboratory, Uppsala University, Uppsala 751 85, Sweden
[7]Division of Genetic and Molecular Toxicology, National Center for Toxicological Research, US Food and Drug Administration, HFT-120, 3900 NCTR Road, Jefferson, AR 72079, USA
[8]MRC Weatherall Institute of Molecular Medicine, University of Oxford, John Radcliffe Hospital, Headington, Oxford OX3 9DS, UK
[9]Lead Contact
*Correspondence: edward.morrissey@imm.ox.ac.uk (E.M.), doug.winton@cruk.cam.ac.uk (D.J.W.)
https://doi.org/10.1016/j.stem.2018.04.020

SUMMARY

We investigated the means and timing by which mutations become fixed in the human colonic epithelium by visualizing somatic clones and mathematical inference. Fixation requires two sequential steps. First, one of approximately seven active stem cells residing within each colonic crypt has to be mutated. Second, the mutated stem cell has to replace neighbors to populate the entire crypt in a process that takes several years. Subsequent clonal expansion due to crypt fission is infrequent for neutral mutations (around 0.7% of all crypts undergo fission in a single year). Pro-oncogenic mutations subvert both stem cell replacement to accelerate fixation and clonal expansion by crypt fission to achieve high mutant allele frequencies with age. The benchmarking of these behaviors allows the advantage associated with different gene-specific mutations to be compared irrespective of the cellular mechanisms by which they are conferred.

INTRODUCTION

The extent to which the cellular properties of adult stem cells determine the risk of neoplastic transformation is currently debated (Tomasetti and Vogelstein, 2015; Wu et al., 2016; Tomasetti et al., 2017). The rationale is that stem cells, once mutated, allow variants to become fixed and subsequently spread within the tissue. However, the fates of individual stem cells in the renewing epithelia most at risk of developing cancers are stochastic (Blanpain and Fuchs, 2014). Consequently, mutation of an individual stem cell establishes unknown probabilities for variant fixation and the rate of lateral clonal expansion.

For colorectal cancers, the conventional view that successive clonal sweeps populate tumors during progression has been called into question. Regional sampling within individual cancers has revealed that subclones are distributed throughout the cancer suggesting that cancers arise as a single expansion event when a combination of factors achieves a critical threshold (Sottoriva et al., 2015). These new concepts make establishing the cellular mechanisms by which somatic variants arise, become fixed and spread within adult colonic epithelium more urgent. To date, these processes have been considered qualitatively, in isolation and not integrated to establish how variant burden accumulates. Consequently, there is no benchmark against which to compare the impact of advantaged or pro-oncogenic mutations.

Previously by analysis of age-related changes in clone frequencies we inferred the stem cell dynamics that dictate the probability of clone fixation in mice (Kozar et al., 2013). Here, the approach is applied to the human colonic epithelium by detecting spontaneous gene-specific mutation. We find that human colonic crypts are maintained by approximately seven clonogenic stem cells of which one is replaced around once every 9 months. Variant fixation requires all wild-type stem cells to be displaced defining a process of monoclonal conversion of crypts that takes many years. Subsequent expansion of neutral clones into multiple crypts by crypt fission is a rare event in adult life. Biased behaviors are confirmed to subvert these processes to achieve variant over representation.

RESULTS

Detection and Analysis of a Known Clonal Mark: mPAS

Few visualizable clonal marks have been described. One previously used in human colon detects loss of O-acetylation of sialomucins using a mildly reductive periodic acid Schiff technique (mPAS) (Veh et al., 1982). mPAS staining of FFPE colon sections from samples obtained at surgical resection confirmed

 © 2018 The Authors. Published by Elsevier Inc.
This is an open access article under the CC BY license (http://creativecommons.org/licenses/by/4.0/).

previously described staining patterns (Sugihara and Jass, 1986). These are composed of crypts that are wholly populated (WPC) or partially populated (PPC) with mPAS$^+$ clones and also crypts containing single positive cells (Figures 1A–1D).

The mode of inheritance of this unknown polymorphic locus indicates that around 90% of the Western population are permissive high O-acetylators (Fuller et al., 1990). The 10% of low-acetylator homozygote individuals are readily identifiable by mPAS$^+$ staining throughout the sample. The permissive high O-acetylators divide into 55.6% uninformative homozygotes and 44.4% that are heterozygotes (Campbell et al., 1994). An image analysis pipeline was developed to detect mPAS$^+$ clones (Figure S1).

Identification and Scoring of mPAS$^+$ Clones

Histologically normal colonic epithelium from surgically resected samples was evaluated for mPAS detection. Of 187 patients (Table S1), 50 ranging between 37 and 93 years of age were informative using defined inclusion criteria. An age-related increase in WPCs was observed (Figure 1E). The slope, ΔC_{fix}, describing accumulation of WPCs, was 5.85×10^{-6} crypts per year (95% margin of error [ME]: $\pm$ 2.68×10^{-6}) (Figure 1F).

Importantly, as expected there was no age-related increase in PPC (Figure 1E), present at around 1.05 per 10^4 crypts (>95% ME: $\pm$ 0.32 per 10^4 crypts). The *de novo* appearance of transition-form PPCs is balanced by their loss due either to stem cell extinctions or expansions that generate WPCs and thereby maintain ΔC_{fix} (Kozar et al., 2013).

The rate of conversion of PPCs (C_{part}) to maintain the slope of ΔC_{fix} indicates that monoclonal conversion of human colonic crypts takes many years (13 years for 90% conversion, median 6.3 years). Notably, this is in accord with observations in patients one year after radiation therapy that clones are predominately PPCs and with published times to monoclonality that are of the order of years (Campbell et al., 1996; Yatabe et al., 2001; Kim and Shibata, 2002).

Determination of *De Novo* Mutation Rate

It is known that both ΔC_{fix} and C_{part} are dependent on the *de novo* mutation rate (Kozar et al., 2013). New mutations can be identified as clones arising in the proliferative zone above but not connected to the crypt base. To determine the mutation rate serial sections from nine patients were stained for mPAS. From 232 tissue sections, containing two million crypts a total of 60 new clones were identified (Figures 1G, 1H, and S2A).

The mutation rate is directly derived from the ratio of the number of positive cells and the total target population (Kozar et al., 2013); in this case, single mPAS$^+$ cells/total goblet cells were estimated (Figures S2B–2D). The selected patients were representative in terms of number of mPAS$^+$ WPC and PPCs (Figure S2E). Variation in estimates across patients may indicate a potential distribution of mutation rates. There was no appreciable age-related trend (Figure 1I). The overall *de novo* mutation rate was 4.44×10^{-6} mutations per mitosis (>95% ME: $\pm$ 2.69×10^{6}).

Inference of Stem Cell Number and Replacement Rate

Combining the estimate of ΔC_{fix} and C_{part} for mPAS$^+$ clones, together with the *de novo* mutation rate, the values for the number of stem cells per crypt (N_{crypt}) and rate of stem cell replacement (λ_{crypt}) were calculated. This revealed that human colonic crypts each contain between 5 and 10 active stem cells (95% Credible Interval (CI); mean = 7). The replacement rate is between 0.65 and 2.7 stem cell replacements/crypt/year (95% CI; mean = 1.3) (Figure 1J). The latter estimate contrasts to the mouse where the replacement rate is nearly 100-fold faster (Kozar et al., 2013).

Validation using New Clonal Marks

To validate the above additional clonal marks were sought. Four genes encoded on the X chromosome, subject to X-inactivation and not associated with DNA repair or pro-oncogenic processes were assessed (Table S2). Antibody staining patterns consistent with truncating mutations were only observed for *MAOA* with both WPC and PPC crypts identified (Figures 2A and 2B). Confirmation of the ability to detect MAOA protein was shown using two independent antibodies in serial sections (Figure 2A). Next, patients were screened to assess the age-related change in MAOA$^-$ clone frequencies (Figures 2C and 2D). As for mPAS an age-related accumulation of WPC and constant background frequency of the transition-form PPCs was observed (Figure 2C). The regression revealed a ΔC_{fix} of 1.76×10^{-6} per year (>95% ME: $\pm$ 0.42×10^{-6}) (Figure 2D).

Rates of clone fixation will vary for different clonal marks because different loci will have different somatic mutation rates. However, the balanced loss/replacement of stem cells that acts to resolve PPCs and support ΔC_{fix} will be identical for neutral marks. Thus $\Delta C_{fix}/C_{part}$ is independent of mutation rate and describes the dynamic that leads to monoclonal conversion. The larger cohort of patients scored for clonal loss of MAOA (152 patients) also contained those informative for mPAS (48). We considered the MAOA data derived from mPAS informative and uninformative patients separately. Reassuringly this revealed that the slopes describing the age related accumulation of MAOA-deficient clones, the background frequencies of transition-form clones and $\Delta C_{fix}/C_{part}$ are near identical for the two subgroups (Figures 2E and S3A–S3E).

Importantly, comparing pooled MAOA and mPAS data reveals similar values for $\Delta C_{fix}/C_{part}$ of 7.2×10^{-2} (>95% ME: $\pm$ 2.3×10^{-2}) and 5.6×10^{-2} (>95% ME: $\pm$ 3.2×10^{-2}), respectively (Figure 2E). This shows that, despite the different mutation rates and resultant clone frequencies, the inferred dynamics of stem cell replacement closely correspond (Figure 2F).

Previously analysis of intra-clone size variation within 11 PPC clones has indicated around six functional stem cells/crypt, similar to our estimate, but also a stem cell replacement rate around 100-fold faster than that derived here (Baker et al., 2014). To resolve this disparity, we focused on the implications of the different estimates on the time for crypt monoclonal conversion. Competition between a small number of stem cells undergoing rapid replacement will inevitably result in the population of the crypt by a single clone in 3 weeks. This is not compatible with documented times to monoclonality described for human crypts and observed here (Figures S3F and S3G) (Campbell et al., 1996; Yatabe et al., 2001; Kim and Shibata, 2002). Further slow replacement rates will only infrequently result in stem cell mediated changes in clone size that can be captured due to the rapid tissue turnover of around 3–8 days (Potten et al., 1992; Baker et al., 2014). This suggests that additional

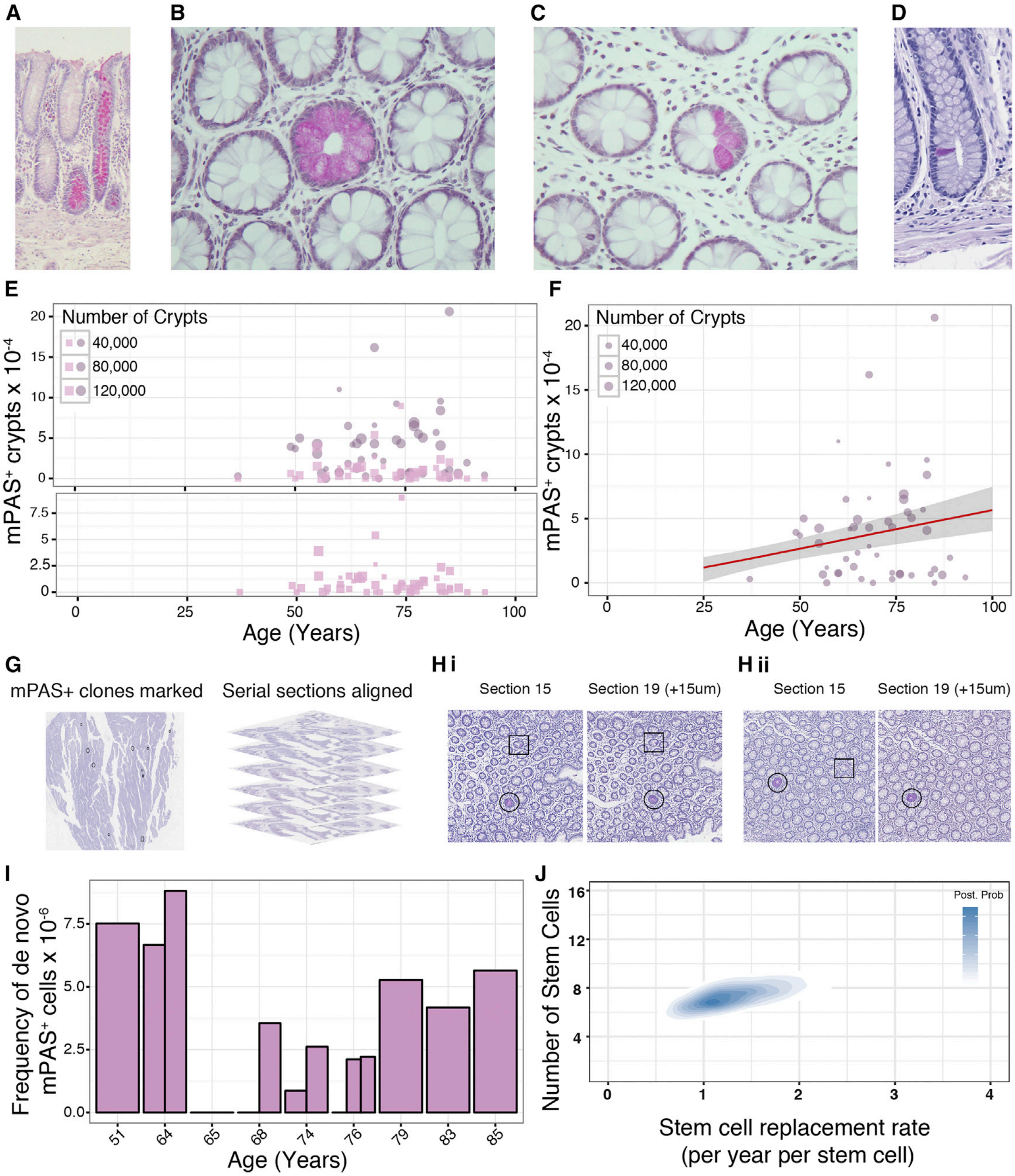

Figure 1. Identification and Quantification of mPAS+ Clones

(A) Longitudinally sectioned sporadic mPAS+ wholly populated crypts (WPC).

(B and C) Sporadic WPC (B) and partially populated crypts (PPC) (C) within *en face* tissue sections.

(D) Single mPAS+ cell within a crypt.

(E) Frequencies of mPAS+ WPC (circles) and PPC (squares) plotted against patient age. Bottom panel shows PPC only on expanded y axis.

(F) Regression analysis showing ΔC_{fix} plotted in red at 5.85×10^{-6} per year with 95% ME in gray.

(G) mPAS+ clones are marked within processed images in black before serial sections are aligned to enable tracking of clones.

(H) WPC (circles) and PPC (squares) can be traced through aligned serial sections (i), while *de novo* mutations occurring in TA cells cannot (ii).

(I) Frequency of *de novo* mPAS+ cells derived for 9 patients plotted by age. Each bar represents a single sample, up to three samples were analyzed per patient. The overall mutation rate (α) was calculated to be 4.44×10^{-6} mutations per mitoses ($\pm 2.69 \times 10^{6}$; ME 95%).

(J) Heatmap representing posterior probabilities for the indicated combination of functional stem cell number for crypt (N, y axis) and the rate of stem cell replacement per year per stem cell (λ, x axis). Colors represent posterior probability, white indicating a very low probability that this value underlies the actual dynamics observed, blue indicating a high likelihood. Inference of N and λ in human colonic crypts indicates between 5 and 10 (95% CI; mean = 7) functional stem cells replacing each other at a rate of between 0.65 and 2.7 stem cell replacements per crypt per year (95% CI; mean = 1.3).

See also Figures S1 and S2.

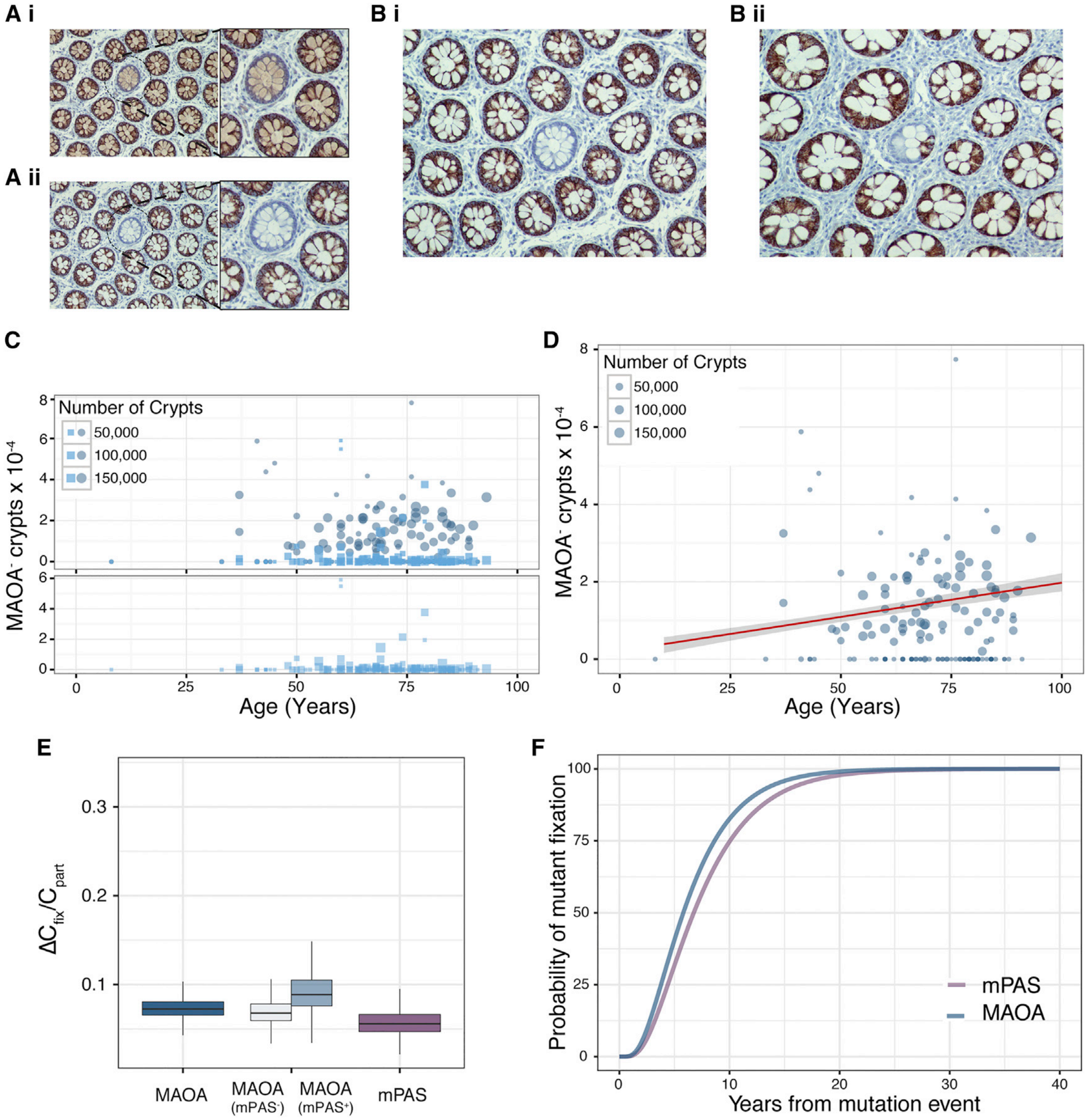

Figure 2. Validation of Clone Dynamics using Novel Clonal Marks

(A) Serial sections (i) and (ii) stained with different antibodies for MAOA. Negative crypt highlighted and enlarged.

(B) (i) WPC and (ii) PPC in *en face* tissue sections stained for MAOA.

(C) Frequency plots of WPC (circles) and PPC (squares) for MAOA^{-} clones for 152 patients (age 8–93 years). Bottom panels show PPC frequencies alone on expanded y axis.

(D) Regression analysis showing ΔC_{fix} for MAOA (1.76×10^{-6} per year) plotted in red with 95% ME shaded in gray.

(E) Boxplot showing similar ratio for $\Delta C_{fix}/C_{part}$ for MAOA ($7.2 \pm 2.3 \times 10^{-2}$) and mPAS ($5.6 \pm 3.2 \times 10^{-2}$) (MAOA mPAS^{-} [$6.8 \pm 2.9 \times 10^{-2}$] and MAOA mPAS+ [$8.9 \pm 4.5 \times 10^{-2}$]). >95% ME.

(F) Inferred mutant fixation times by crypt monoclonal conversion plotted using parameters derived from spontaneous mPAS^{+} and MAOA^{-} clones.

See also Figure S3.

processes such as variation in the number of amplifying cell divisions and/or variation in the extent of lateral versus vertical migration of transit amplifying cells contribute to the fluctuations in clone size as progeny move toward the luminal surface.

Identifying Biased Behaviors

To establish whether analysis of clone dynamics has the potential to identify advantage for potentially pro-oncogenic mutations a further four genes (*APEX2*, *POLA1*, *RBBP4*, and *STAG2*) encoded on the X chromosome and associated with DNA repair or pro-oncogenic function were assessed (Table S2). Staining consistent with clonal truncating mutations was identified for *STAG2* only (Figures 3A and 3B). This was confirmed with two independent antibodies (Figure 3A). STAG2^{-} WPC and PPC were observed (Figure 3B). The former showed an age-related increase (Figure 3C) and ΔC_{fix} determined to be 1.96×10^{-5}

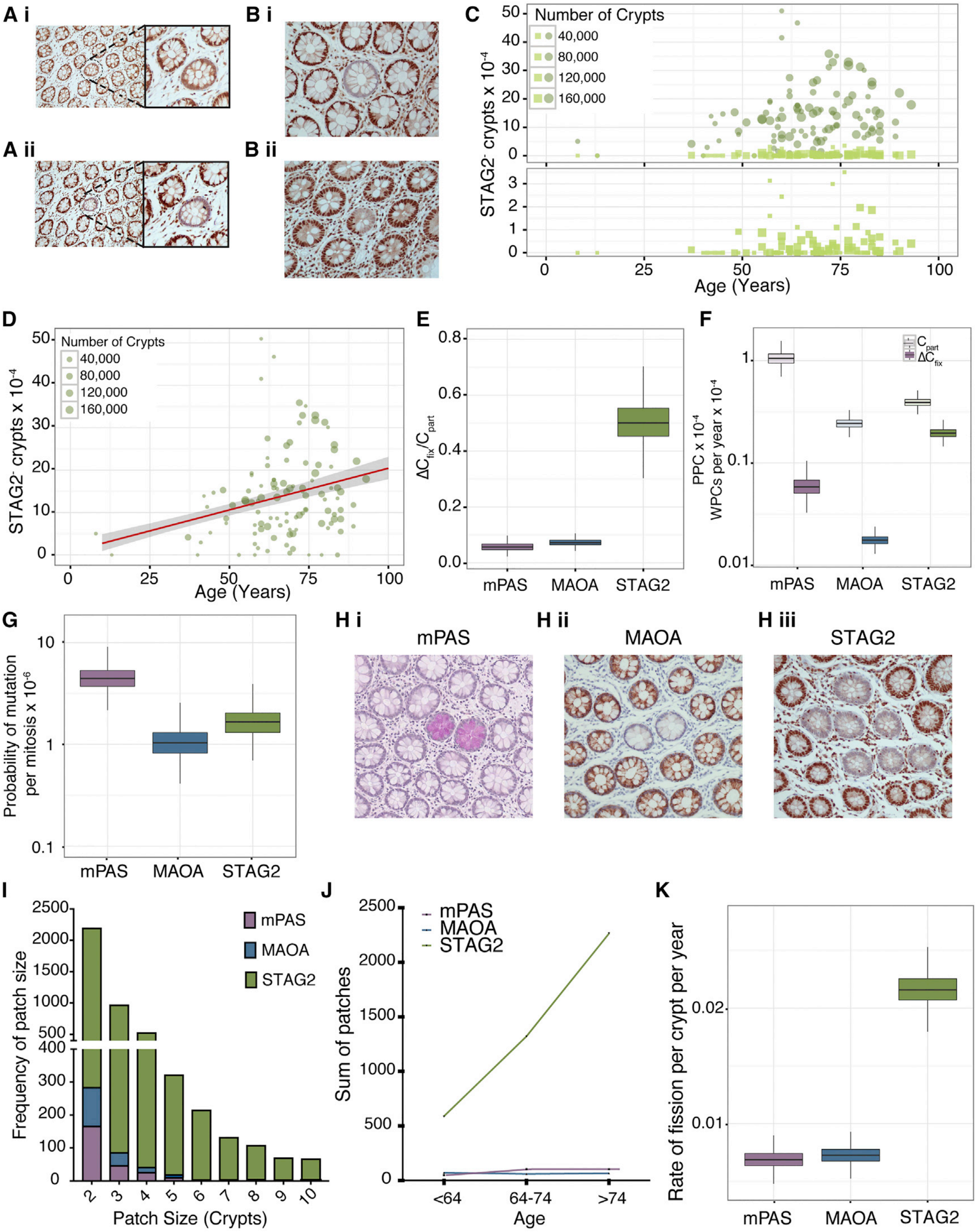

Figure 3. Comparison of Marks Reveals Bias for STAG2 Mutation

(A) Serial sections (i) and (ii) stained with different antibodies for STAG2. Negative crypt highlighted and enlarged.

(B) (i) WPC and (ii) PPC in *en face* tissue sections stained for STAG2.

(C) Frequency plots of WPC (circles) and PPC (squares) for STAG2-deficient clones for 186 patients (age 8–93 years). Bottom panels show PPC frequencies only on expanded y axis.

(D) Regression analysis showing ΔC_{fix} for STAG2 (1.96×10^{-5} per year) plotted in red with 95% ME shaded in gray.

(E) Boxplot showing similar ratio for $\Delta C_{fix}/C_{part}$ for MAOA and mPAS ($7.2 \pm 2.3 \times 10^{-2}$ and $5.6 \pm 3.2 \times 10^{-2}$) while STAG2 shows 10× increased ratio at (50×10^{-2}; >95% ME: $\pm$ 14 $\times 10^{-2}$).

(F) Boxplot showing ΔC_{fix} (dark boxes) and C_{part} (light boxes) for the three clonal marks. >95% ME.

(G) Boxplot showing the calculated mutation rate for each clonal mark. >95% ME.

(H) Histological sections showing multicrypt patches for (i) mPAS^{+}, (ii) MAOA^{-}, and (iii) STAG2^{-} crypts.

(legend continued on next page)

per year (>95% ME: ± 0.42 × 10^{-5}) (Figure 3D). Notably the ratio $\Delta C_{fix}/C_{part}$ was increased 10-fold (50 × 10^{-2}; >95% ME: ± 14 × 10^{-2}) compared to that observed for MAOA and mPAS (Figure 3E) and arises due to an under-representation of PPCs (Figure 3F), suggesting a bias in stem cell replacement. Applying the values for N_{crypt} and λ_{crypt} determined above (7 and 1.3/year, respectively), we estimate that this probability departs from neutral replacement (0.5) to around 0.99 (95% CI: 0.8–0.99), i.e., near certainty that a STAG2-deficient stem cell will populate the crypt. Consequently, the time for monoclonal conversion is reduced and most mutant clones become fixed.

Direct versus Indirect Effect of *STAG2* Mutation

STAG2 encodes a subunit of the cohesin complex, has been associated with aneuploidy, and is a tumor suppressor gene (Kim et al., 2012; Hill et al., 2016). STAG2 loss results in prolonged association of telomeric repeats during the cell cycle and that this may result in genomic rearrangements (Daniloski and Smith, 2017). To explore whether the biased behavior of STAG2-deficient stem cells arises directly or whether it could be mediated by subsequent elevated genomic instability, we performed simulations. These allow a first neutral mutation (*STAG2*) followed by a second higher probability mutation conferring advantage (certain to replace wild-type neighbors) that drives the altered clone dynamics. This was compared to the principal mathematical model, which simulates a single altering mutation. The comparison revealed that the rate of second mutation has to be increased by the order of 10^5 before all clones contain both mutations and that even this level of hypermutation fails to explain the observed age related increase in STAG2-deficient clones (Figures S3H and S3I). To impact on intracryptal clone dynamics secondary mutations conferring advantage have to occur in *STAG2* mutant clones while they are still PPCs and this requires an extremely high mutation rate. It is likely the clone dynamics described arise directly from STAG2 loss.

Inference of *MAOA* and *STAG2* Mutation Rates

Using the ratio of C_{part} between mPAS and MAOA and the mutation rate derived for mPAS, we can estimate the mutation rate for *MAOA* to be 1.03 × 10^{-6} mutations per mitosis (>95% ME: 1.03 ± 0.78 × 10^{-6}). As *STAG2* mutation is not neutral, we use the full equations taking N = 7 and P_R = 0.99 leading to a mutation rate for *STAG2* of 1.66 × 10^{-6} mutations per mitosis (>95% ME: 1.66 ± 1.3 × 10^{-6}) (Figure 3G). Notably the X-linked gene *PIGA* that forms the basis for mutagenesis screens and that is of similar size and intron/exon structure to *MAOA* has a comparable mutation rate in human cells of 10^{-6}/mitosis (Araten et al., 2005).

Clonal Expansion beyond the Crypt

Colonic clones can expand beyond individual glands by crypt fission (Greaves et al., 2006). For all three clonal marks, patches of mutant epithelium comprising two or more crypts were observed at a low frequency (Figure 3H). Patches of two were frequently identified for mPAS$^+$ and MAOA$^-$ marked crypts, whereas larger patches were uncommon. Larger patches were frequently observed for STAG2$^-$ crypts that also showed an age-related increase (Figures 3I and 3J).

The age-related change in the patch sizes was modeled. This revealed the crypt fission rate for mPAS$^+$ and MAOA$^-$ crypts to be 0.68% (95% CI: 0.68 ± 0.15) and 0.72% (95% CI: 0.72 ± 0.15) per year, respectively. STAG2-deficient crypts undergo fission at a rate of 2.15% (95% CI: 2.15 ± 0.27) per year (Figure 3K), 3-fold that of normal fission rates and accounting for the larger patches observed. Therefore, as well as conferring an advantage to the stem cells within the crypt, STAG2 deficiency also enables lateral expansion to generate large patches within the epithelium.

Previous estimates for the rate of colonic crypt fission have employed different approaches and have varied widely with estimates ranging between 3% and 22% of crypts undergoing fission per year (Totafurno et al., 1987; Baker et al., 2014). The lower estimate of 0.7% derived here is consistent with other studies documenting age-related changes in genomic methylation patterns that found no conserved patterns between neighboring crypts, suggesting most crypts survive without undergoing fission during adult life (Kim and Shibata, 2002, 2004).

Recently a crypt fusion process has been described in mouse intestinal epithelium at a rate equivalent to that of fission (Bruens et al., 2017). Mutant fusion has two possible outcomes. Either a mutant crypt can fuse with another mutant crypt thereby reducing patch size or it can fuse with a wild-type crypt in which case the patch size could reduce or stay the same. To determine whether fusion could impact on our interpretation of clone size data, we used stochastic simulations to explore the effect of different fusion rates (Figure S4). This analysis showed that the patch size is dominated by crypt fission with fusion having a negligible effect in comparison. This was observed for both the neutral and advantageous mutations.

Implications for Fixation and Spread of Clonal Variants

The accumulated burden of neutral somatic variants within the human colonic epithelium varies with the mutation rate (Figure 4A). However, selection can act either to promote fixation of mutant clones by biased stem cell replacement and/or to promote their spread by elevated rates of crypt fission. For example, an increase in the probability of variant stem cell replacement (from 0.5 to 0.99) alone increases the burden of somatic variants 7-fold. Similarly, promoting the generation of larger patches by elevated fission rate alone would increase variant burden 2-fold. Together a 14-fold increase by age 60 results (Figure 4B).

Increased fission rates may contribute to field cancerization effects, whereby an area of histologically normal epithelium containing genetic alterations predisposes to neoplastic development (Braakhuis et al., 2003). Such effects have been described

(I) Histogram showing patch sizes for mPAS, MAOA, and STAG2.

(J) Plot showing an age-associated increase in number of patches.

(K) Boxplot showing the inferred crypt fission rate for each of the clonal marks, with rates for mPAS$^+$ and MAOA$^-$ crypts of around 0.68% (95% CI: 0.68 ± 0.15) and 0.72% (95% CI: 0.72 ± 0.15) per year. However, STAG2-deficient crypts undergo fission at a rate of 2.15% (95% CI: 2.15 ± 0.27) per year.

See also Figure S3.

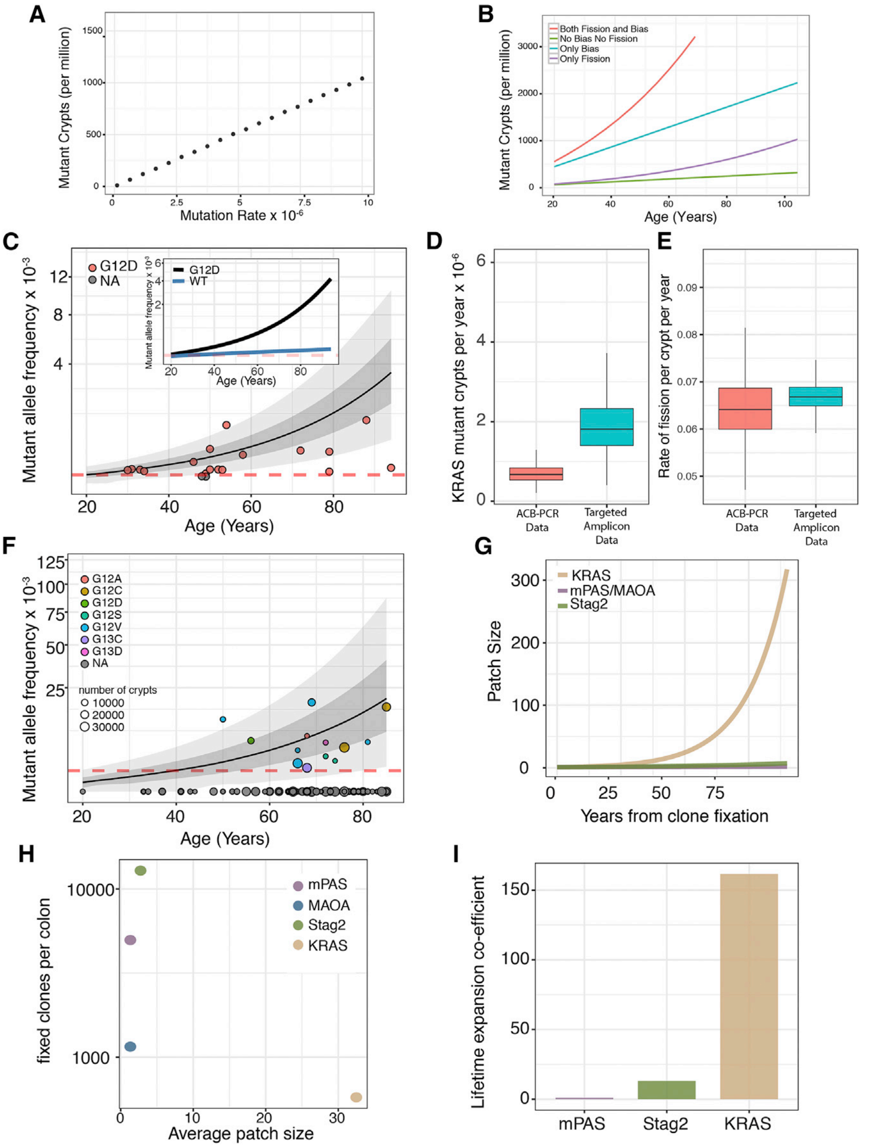

Figure 4. Expansion Coefficient Predicts Age-Related Mutation Burden

(A) Simulation demonstrating mutation rate determines accumulated mutation burden at age 60 years for neutral genes.

(B) Simulated mutation burden of the colon plotted against patient age for notional genes sharing a common mutation rate (2×10^{-6}/mitosis). Plots show neutral outcome (green), mutation conferring increased P_R (0.99) only (blue), mutation conferring 3-fold increase in rate of fission only (purple), and mutations conferring both increased 3-fold fission rate and P_R of 0.99 (red) that corresponds to the observed consequence of STAG2 mutation.

(C) Mutant allele frequency data of *KRAS*(G12D) mutations from 20 patients determined using allele-specific competitive blocker (ACB)-PCR method. Patient data are represented by red circles. The mean (black line) and 95% CI (grayed area) of the model is shown. Red dotted line shows detection threshold. Inset demonstrating the contribution of fission shows the predicted average accumulation of *KRAS* (G12D) mutant alleles with inferred elevated (black) and wild-type (blue) fission rates, respectively.

(D) Boxplot to show the accumulation of *KRAS* mutant crypts using both the ACB-PCR method and targeted amplicon sequencing on a separate set of patients. >95% ME.

(E) Boxplot to show percentage of *KRAS* mutant crypts undergoing fission per year using both the ACB-PCR and targeted amplicon data. >95% ME.

(F) Mutant allele frequency data of *KRAS* mutations from 126 individuals plotted against age. 13 individuals displayed detectable mutations, and the mean accumulation of mutant allele calculated using the model is plotted (black line) as well as the 95% CI. Red dotted line shows detection threshold.

(G) The calculated patch size of crypts mutant in respect of *KRAS*, *STAG2*, or *MAOA*/mPAS shows a significant expansion of KRAS mutant patches in the human colon following clone fixation.

(H) The average patch size of each clonal mark plotted against the number of fixed clones per colon shows a small number of small clones for MAOA, with similar patch sizes but higher in frequency for mPAS and STAG2. While for *KRAS* a small number of large patches is predicted.

(I) Lifetime coefficient of expansion normalized to a neutral mark (mPAS shown) allows comparison of relative advantage.

See also Figure S4.

in the context of Crohn's ileocolitis (Galandiuk et al., 2012) and sporadic colorectal cancer (Shen et al., 2005).

Clone Dynamics and Kras Mutation

The rate of oncogene activation by single nucleotide mutation is several orders of magnitude lower than the loss-of-function mutations described here. For example, the G > A transversion that creates the *KRAS*(G12D) occurs at a frequency of around 4.0×10^{-8} per year (Tomasetti et al., 2013). Despite this, *KRAS*(G12D) mutations at high allele frequencies (mutant allele frequency [MAF]) have been described in the normal mucosa including those of patients not known to have cancer (Dieterle et al., 2004; Kraus et al., 2006; Parsons et al., 2010). We sought to identify the changes in stem cell behaviors that could explain such high MAFs.

Initially, we considered the data from one study employing a sensitive competitive PCR-based method that described a MAF of around 1.44×10^{-4} (1/3,500 epithelial cells) for *KRAS*(G12D) mutation in the normal mucosa of 20 patients (Table S3) (Parsons et al., 2010). The stem cell behaviors giving an optimal fit to the data show that mutation and intracryptal fixation rates cannot explain the large range of *KRAS* MAFs, which requires a 10-fold increase in rate of lateral expansion of pre-existing clones (Figure 4C).

In validation, we performed targeted sequencing analysis to detect all activating mutations at *KRAS* for codons 12/13 on

188 individuals. Sections from 13 of 126 patients had detectable MAFs in range of 0.2%–1.8% with an estimated sensitivity of detection of 10^{-3}. Inference of the optimal values for ΔC_{fix} and fission rate gave values similar to those derived from the Parsons dataset above (Figures 4D-4F) and confirm that the observed data can only be explained by a 10-fold increase in the lateral expansion of *KRAS* mutant crypts. To explore how clone sizes change subsequent to clone fixation, simulations were run using the 10-fold elevated fission rate of 7% per annum to show the average patch size obtained over 60 years (Figure 4G). The mutational burden results from the extensive expansion of a small number of somatic clones in contrast to the more modest expansion of the loss-of-function clonal marks described earlier (Figure 4H). Elevated fission rates for *KRAS*(G12D) resulting in enlarged multicrypt patches have been described for murine crypts (Snippert et al., 2014).

Ranking Advantage Conferred by Gene-Specific Mutation

MAFs for different genes do not themselves convey whether selective advantage is conferred, as they largely depend on mutation rate. By normalizing for mutation rate and describing the combined effects of intra-crypt dynamics and subsequent fission over time, a quantitative measure of selective advantage can be extracted that allows different mutation events to be directly compared (Figure 4I). Thus, *KRAS(G12D)* and *STAG2* mutations have an average lifetime expansion coefficient (C_{exp}) that is 155- and 13-fold greater than neutral mutations, respectively (Figure 4I).

DISCUSSION

Previous attempts to model the rate of fixation of somatic mutations in human colonic epithelium have recognized the need to consider physiological stem cell turnover in determining the probability of fixation (Araten et al., 2005; Kang and Shibata, 2013; Tomasetti and Vogelstein, 2015; Wu et al., 2016; Tomasetti et al., 2017). However, the parameters for crucial metrics such as number of functional stem cells and the frequency of stem cell replacement have been lacking. In addition, there has been no consideration of how mutational burden is additionally dictated by crypt fission that allow lateral spreading of variants beyond individual crypts. Together these factors have prevented benchmarking of how age-related mutation burden arises within the colonic epithelium.

The dynamics of clone expansion resulting in monoclonality of human colonic crypts is notably longer than in mouse, taking several years. The precise cellular behaviors that underpin these dynamics are unclear. A paucity of data on stem cell cycle times for the human epithelium makes relating stem cell replacement to the frequency of cell division impossible. In addition, observations in the mouse show that cells positioned lower (center) and higher (border) with respect to the crypt base have different self-renewal probabilities but also frequently exchange between these positions (Ritsma et al., 2014). Thus, the overarching neutral drift dynamic that we and others have described is the resolved behavior of the total stem cell population (Lopez-Garcia et al., 2010; Snippert et al., 2010; Kozar et al., 2013). Given the larger size of the human crypt, such reciprocal exchanges may be more complex, and this is likely to contribute to the slow dynamics of monoclonal conversion.

For neutral mutations, the cumulative colonic MAF scales directly to mutation rate. However, understanding the mutational burden associated with biased behaviors requires a quantitative description of the normal processes that are subverted. Frequent loss-of-function mutations can reach high proportions just by impacting on stem cell replacement processes within the crypt. For example, *STAG2*-deficient stem cells are advantaged in the process of intra-crypt competition and in the subsequent expansion beyond the crypt. It follows that expansions of mutant epithelium arise as the first process increases the numbers of *STAG2*-deficient crypts available for subsequent fission events.

Around half of the somatic mutations present in colorectal cancers are thought to arise in the epithelium before oncogenic transformation (Tomasetti et al., 2013). The expansion of *KRAS*-activating mutations to generate large patch sizes lends itself to this outcome and demonstrates how powerful oncogenes may actively contribute to tumor development through a field cancerization effect. As shown here, this outcome can be described knowing only the rate of mutation and the final MAF.

Here by benchmarking and integrating the relative contributions of mutation rate and cell renewal/expansion processes in dictating age-related mutational burden, we provide a means to express the advantage conferred by gene specific mutations. This will allow different mutations to be compared and ranked for advantage within a common framework irrespective of the specific cellular mechanism by which it is conferred. Practically, these benchmarks define the nature and window of opportunity for chemoprevention to limit expansion of pro-oncogenic mutation and thereby limit cancer risk.

STAR★METHODS

Detailed methods are provided in the online version of this paper and include the following:

- KEY RESOURCES TABLE
- CONTACT FOR REAGENT AND RESOURCE SHARING
- EXPERIMENTAL MODEL AND SUBJECT DETAILS
  - Human tissue
- METHOD DETAILS
  - Mild PAS (mPAS) staining
  - Image segmentation and mPAS clone detection
  - Estimating the mutation rate for the mPAS clonal mark
  - Evaluation of X-linked genes for clonal analysis
  - Immunohistochemistry
  - Targeted amplicon KRAS sequencing
- QUANTIFICATION AND STATISTICAL ANALYSIS
  - Statistical Analysis of clone data
  - Analysis of KRAS sequencing data
- DATA AND SOFTWARE AVAILABILITY

SUPPLEMENTAL INFORMATION

Supplemental Information includes four figures and three tables and can be found with this article online at https://doi.org/10.1016/j.stem.2018.04.020.

ACKNOWLEDGMENTS

The authors thank the Histology and Genomics cores at the CRUK Cambridge Institute for technical support and Prof. Fiona Campbell (Department of Pathology, University of Liverpool) for helpful discussions on the mPAS technique during conceptualization of the project. The authors acknowledge the contribution and support to this research provided by the Norwich Research Park BioRepository (Human Tissue Authority, license number 11208; NRES REC no. 08/H0304/85+5), a facility supported by funding from the BBSRC, the Norfolk and Norwich University Hospitals NHS Foundation Trust, and the University of East Anglia. The authors also acknowledge the contribution and support of Addenbrooke's Human Research Tissue Bank that is supported by the NIHR Cambridge Biomedical Research Centre. Funding was provided for Wellcome Trust Grant (103805), Cancer Research UK, Wellcome four-year Ph.D. studentship, and an MRC Computational Biology Fellowship (MC_UU_12025, MRC Strategic Alliance Funding; MRC Weatherall Institute of Molecular Medicine).

AUTHOR CONTRIBUTIONS

C.O., A.H., A.-S.T., T.R., M.C., and K.M. performed experiments. R.B.-S., P.Q., S.M., R.t.-H., A.I., and M.W. provided patient samples. C.L. provided antibodies. M.B.M. and B.P. provided *KRAS* mutant allele data. S.T. interpreted data. R.K. analyzed experiments and interpreted data. A.M.N. performed experiments and analyzed experiments. E.M. developed image analysis pipelines, performed mathematical modeling, and analyzed experiments. D.J.W. and E.M. conceived, and with A.M.N., designed the study, interpreted data, and with C.O., wrote the manuscript. All authors reviewed the manuscript.

DECLARATION OF INTERESTS

The authors declare no competing interests.

Received: August 25, 2017
Revised: February 16, 2018
Accepted: April 23, 2018
Published: May 17, 2018

REFERENCES

Araten, D.J., Golde, D.W., Zhang, R.H., Thaler, H.T., Gargiulo, L., Notaro, R., and Luzzatto, L. (2005). A quantitative measurement of the human somatic mutation rate. Cancer Res. *65*, 8111–8117.

Baker, A.-M., Cereser, B., Melton, S., Fletcher, A.G., Rodriguez-Justo, M., Tadrous, P.J., Humphries, A., Elia, G., McDonald, S.A.C., Wright, N.A., et al. (2014). Quantification of crypt and stem cell evolution in the normal and neoplastic human colon. Cell Rep. *8*, 940–947.

Blanpain, C., and Fuchs, E. (2014). Stem cell plasticity. Plasticity of epithelial stem cells in tissue regeneration. Science *344*, 1242281.

Braakhuis, B.J.M., Tabor, M.P., Kummer, J.A., Leemans, C.R., and Brakenhoff, R.H. (2003). A genetic explanation of Slaughter's concept of field cancerization: Evidence and clinical implications. Cancer Res. *63*, 1727–1730.

Bradski, G. (2000). The OpenCV library. Doctor Dobbs J. *25*, 120–126.

Bruens, L., Ellenbroek, S.I.J., van Rheenen, J., and Snippert, H.J. (2017). In vivo imaging reveals existence of crypt fission and fusion in adult mouse intestine. Gastroenterology *153*, 674–677.e3.

Campbell, F., Appleton, M.A.C., Fuller, C.E., Greeff, M.P., Hallgrimsson, J., Katoh, R., Ng, O.L.I., Satir, A., Williams, G.T., and Williams, E.D. (1994). Racial variation in the O-acetylation phenotype of human colonic mucosa. J. Pathol. *174*, 169–174.

Campbell, F., Williams, G.T., Appleton, M.A., Dixon, M.F., Harris, M., and Williams, E.D. (1996). Post-irradiation somatic mutation and clonal stabilisation time in the human colon. Gut *39*, 569–573.

Carpenter, B., Gelman, A., Hoffman, M., Lee, D., Goodrich, B., Betancourt, M., Brubaker, M., Guo, J., and Li, P.R.A. (2017). Stan: A probabilistic programming language. J. Stat. Softw. *76*, 1–32.

Daniloski, Z., and Smith, S. (2017). Loss of tumor suppressor STAG2 promotes telomere recombination and extends the replicative lifespan of normal human cells. Cancer Res. *77*, 5530–5542.

Dieterle, C.P., Conzelmann, M., Linnemann, U., and Berger, M.R. (2004). Detection of isolated tumor cells by polymerase chain reaction-restriction fragment length polymorphism for K-ras mutations in tissue samples of 199 colorectal cancer patients. Clin. Cancer Res. *10*, 641–650.

Fuller, C.E., Davies, R.P., Williams, G.T., and Williams, E.D. (1990). Crypt restricted heterogeneity of goblet cell mucus glycoprotein in histologically normal human colonic mucosa: A potential marker of somatic mutation. Br. J. Cancer *61*, 382–384.

Galandiuk, S., Rodriguez-Justo, M., Jeffery, R., Nicholson, A.M., Cheng, Y., Oukrif, D., Elia, G., Leedham, S.J., McDonald, S.A.C., Wright, N.A., and Graham, T.A. (2012). Field cancerization in the intestinal epithelium of patients with Crohn's ileocolitis. Gastroenterology *142*, 855–864.e8.

Goode, A., Gilbert, B., Harkes, J., Jukic, D., and Satyanarayanan, M. (2013). OpenSlide: A vendor-neutral software foundation for digital pathology. J. Pathol. Inform. *4*, 27.

Greaves, L.C., Preston, S.L., Tadrous, P.J., Taylor, R.W., Barron, M.J., Oukrif, D., Leedham, S.J., Deheragoda, M., Sasieni, P., Novelli, M.R., et al. (2006). Mitochondrial DNA mutations are established in human colonic stem cells, and mutated clones expand by crypt fission. Proc. Natl. Acad. Sci. USA *103*, 714–719.

Hill, V.K., Kim, J.-S., and Waldman, T. (2016). Cohesin mutations in human cancer. Biochim. Biophys. Acta *1866*, 1–11.

Kang, H., and Shibata, D. (2013). Direct measurements of human colon crypt stem cell niche genetic fidelity: The role of chance in non-darwinian mutation selection. Front. Oncol. *3*, 264.

Kim, K.-M., and Shibata, D. (2002). Methylation reveals a niche: Stem cell succession in human colon crypts. Oncogene *21*, 5441–5449.

Kim, K.-M., and Shibata, D. (2004). Tracing ancestry with methylation patterns: Most crypts appear distantly related in normal adult human colon. BMC Gastroenterol. *4*, 8.

Kim, M.S., Kim, S.S., Je, E.M., Yoo, N.J., and Lee, S.H. (2012). Mutational and expressional analyses of STAG2 gene in solid cancers. Neoplasma *59*, 524–529.

Kozar, S., Morrissey, E., Nicholson, A.M., van der Heijden, M., Zecchini, H.I., Kemp, R., Tavaré, S., Vermeulen, L., and Winton, D.J. (2013). Continuous clonal labeling reveals small numbers of functional stem cells in intestinal crypts and adenomas. Cell Stem Cell *13*, 626–633.

Kraus, M.C., Seelig, M.H., Linnemann, U., and Berger, M.R. (2006). The balanced induction of K-ras codon 12 and 13 mutations in mucosa differs from their ratio in neoplastic tissues. Int. J. Oncol. *29*, 957–964.

Lopez-Garcia, C., Klein, A.M., Simons, B.D., and Winton, D.J. (2010). Intestinal stem cell replacement follows a pattern of neutral drift. Science *330*, 822–825.

Martinez, K.C.J. (2005) VIPS—A highly tuned image processing software architecture. In Proceedings of IEEE International Conference on Image Processing, pp. 574–577.

Masella, A.P., Bartram, A.K., Truszkowski, J.M., Brown, D.G., and Neufeld, J.D. (2012). PANDAseq: Paired-end assembler for illumina sequences. BMC Bioinformatics *13*, 31.

Parsons, B.L., Marchant-Miros, K.E., Delongchamp, R.R., Verkler, T.L., Patterson, T.A., McKinzie, P.B., and Kim, L.T. (2010). ACB-PCR quantification of K-RAS codon 12 GAT and GTT mutant fraction in colon tumor and non-tumor tissue. Cancer Invest. *28*, 364–375.

Potten, C.S., Kellett, M., Roberts, S.A., Rew, D.A., and Wilson, G.D. (1992). Measurement of in vivo proliferation in human colorectal mucosa using bromodeoxyuridine. Gut *33*, 71–78.

Ritsma, L., Ellenbroek, S.I.J., Zomer, A., Snippert, H.J., de Sauvage, F.J., Simons, B.D., Clevers, H., and van Rheenen, J. (2014). Intestinal crypt homeostasis revealed at single-stem-cell level by in vivo live imaging. Nature *507*, 362–365.

Ruifrok, A.C., and Johnston, D.A. (2001). Quantification of histochemical staining by color deconvolution. Anal. Quant. Cytol. Histol. *23*, 291–299.

Shen, L., Kondo, Y., Rosner, G.L., Xiao, L., Hernandez, N.S., Vilaythong, J., Houlihan, P.S., Krouse, R.S., Prasad, A.R., Einspahr, J.G., et al. (2005). MGMT promoter methylation and field defect in sporadic colorectal cancer. J. Natl. Cancer Inst. *97*, 1330–1338.

Snippert, H.J., van der Flier, L.G., Sato, T., van Es, J.H., van den Born, M., Kroon-Veenboer, C., Barker, N., Klein, A.M., van Rheenen, J., Simons, B.D., and Clevers, H. (2010). Intestinal crypt homeostasis results from neutral competition between symmetrically dividing Lgr5 stem cells. Cell *143*, 134–144.

Snippert, H.J., Schepers, A.G., van Es, J.H., Simons, B.D., and Clevers, H. (2014). Biased competition between Lgr5 intestinal stem cells driven by oncogenic mutation induces clonal expansion. EMBO Rep. *15*, 62–69.

Sottoriva, A., Kang, H., Ma, Z., Graham, T.A., Salomon, M.P., Zhao, J., Marjoram, P., Siegmund, K., Press, M.F., Shibata, D., and Curtis, C. (2015). A Big Bang model of human colorectal tumor growth. Nat. Genet. *47*, 209–216.

Sugihara, K., and Jass, J.R. (1986). Colorectal goblet cell sialomucin heterogeneity: Its relation to malignant disease. J. Clin. Pathol. *39*, 1088–1095.

Tomasetti, C., and Vogelstein, B. (2015). Cancer etiology. Variation in cancer risk among tissues can be explained by the number of stem cell divisions. Science *347*, 78–81.

Tomasetti, C., Vogelstein, B., and Parmigiani, G. (2013). Half or more of the somatic mutations in cancers of self-renewing tissues originate prior to tumor initiation. Proc. Natl. Acad. Sci. USA *110*, 1999–2004.

Tomasetti, C., Li, L., and Vogelstein, B. (2017). Stem cell divisions, somatic mutations, cancer etiology, and cancer prevention. Science *355*, 1330–1334.

Totafurno, J., Bjerknes, M., and Cheng, H. (1987). The crypt cycle. Crypt and villus production in the adult intestinal epithelium. Biophys. J. *52*, 279–294.

Veh, R.W., Meessen, D., and Kuntz, D.M.B. (1982). Colonic Carcinogenesis (Lancaster Press).

Vermeulen, L., Morrissey, E., van der Heijden, M., Nicholson, A.M., Sottoriva, A., Buczacki, S., Kemp, R., Tavaré, S., and Winton, D.J. (2013). Defining stem cell dynamics in models of intestinal tumor initiation. Science *342*, 995–998.

Wu, S., Powers, S., Zhu, W., and Hannun, Y.A. (2016). Substantial contribution of extrinsic risk factors to cancer development. Nature *529*, 43–47.

Yatabe, Y., Tavaré, S., and Shibata, D. (2001). Investigating stem cells in human colon by using methylation patterns. Proc. Natl. Acad. Sci. USA *98*, 10839–10844.

STAR★METHODS

KEY RESOURCES TABLE

REAGENT or RESOURCE	SOURCE	IDENTIFIER
Antibodies		
Mouse monoclonal anti-MAOA	Santa Cruz Biotechnology	Sc-271123; RRID: AB_10609510
Rabbit polyclonal anti-MAOA	Sigma	HPA059299; RRID: AB_2683970
Goat polyclonal anti-STAG2	LifeSpan BioSciences	LS-B11284; RRID: AB_2725802
Rabbit polyclonal anti-STAG2	Sigma	HPA002857; RRID: AB_1079861
Biological Samples		
Normal human colon FFPE blocks	Addenbrooke's Hospital Cambridge and Norfolk and Norwich University Hospital	Ethical approval 06/Q0108/307 and 08/H0304/85
Critical Commercial Assays		
QIAamp DNA FFPE tissue kit	QIAGEN	56404
Deposited Data		
Human reference genome NCBI build 38, GRCh38.p7	Genome Reference Consortium	https://www.ncbi.nlm.nih.gov/projects/genome/assembly/grc/human/
KRAS amplicon sequencing data (Illumina)	This paper	NCBI Study Accession SRP139051
Oligonucleotides		
Primer Kras Exon 2 forward: ACACT GACGACATGGTTCTACA-GGTGGA GTATTTGATAGTGTATTAACC	This paper	N/A
Primer Kras Exon 2 reverse: TACGG TAGCAGAGACTTGGTCT-TAGCTGT ATCGTCAAGGCAC	This paper	N/A
Software and Algorithms		
PANDAseq 2.11	Masella et al. 2012	https://github.com/neufeld/pandaseq/releases
Frequency of nucleotide calculation PERL scripts	This paper	https://github.com/keke05/KRAS-sequencing/blob/bd775fc005f89198116a8be97531bd9ec5f5f5ca/NUCLEOTIDE_COUNT_FOR_HASH.pl https://github.com/kemp05/KRAS-sequencing/blob/bd759fc005f89198116a8be97531bd9ec5f5f5ca/HASH_3.pl
Image segmentation of crypts and clones: DeCryptICS algorithm	Manuscript in preparation	https://github.com/MorrisseyLab/DeCryptICS
Zegami image collection management	N/A	https://zegami.com/
Google maps pathology viewer	N/A	https://iime.github.io/virtualmicroscope/
Crypt stochastic drift software: CryptDriftR	Manuscript in preparation	https://github.com/MorrisseyLab/CryptDriftR

CONTACT FOR REAGENT AND RESOURCE SHARING

Further information and requests for resources and reagents should be directed to and will be fulfilled by the Lead Contact, Douglas J. Winton (doug.winton@cruk.cam.ac.uk).

EXPERIMENTAL MODEL AND SUBJECT DETAILS

Human tissue

Normal colon tissue samples were collected from both Addenbrooke's Hospital Cambridge and Norfolk and Norwich University Hospital under full ethical approval (06/Q0108/307 and 08/H0304/85 respectively) according to UK Home Office regulations. A total

of 187 patients were included in the study with an age range of 8-93 years (Table S1). Colectomy specimens were fixed in 10% neutral buffered formalin and from areas of tissue clear of any disease, mucosal sheets were stripped from the specimens and embedded *en* face in paraffin blocks.

METHOD DETAILS

Mild PAS (mPAS) staining

From each sample 5 um sections were cut and mounted onto charged slides. Sections were de-waxed and rehydrated before washing in 0.1 M Acetate buffer pH 5.5 at 4 degrees for 5 minutes. Sections were then oxidised in 1 mM sodium periodate buffer at 4°C for 10 minutes before washing in 1% glycerol for 5 minutes. Three washes were performed in ultra-pure water for 5 minutes in total before sections were stained in Schiff's reagent for 15 minutes. Sections were washed again in ultra-pure water before counter-staining in Mayer's Haematoxylin for 40 s. Finally sections were washed again in ultra-pure water, blued briefly in tap water before rinsing in ultra pure water prior to dehydration, clearing and mounting in DPX.

Image segmentation and mPAS clone detection

Sections from all blocks were stained using mPAS and manually viewed to determine stain quality. Each section was scanned using Aperio software and an image analysis algorithm was devised that identified the number of crypts and the position of mPAS$^+$ clones within each stained section (Figure S1). In order to be confident that patients included were informative heterozygotes, an inclusion criterion of > 7000 crypts and at least one sporadic clone detected were set.

Algorithm overview

The aim of the image processing was to both find rare clones (~1 in 10,000 crypts) highlighted by the chosen clonal mark, as well as identify all crypts along with their sizes and shape parameters. The tissue images are gigapixel in size, typically of the order of 50,000 × 50,000 pixels.

Briefly, the algorithm first splits the image into smaller tiles of size 20,000 × 20,000, it then employs color deconvolution on the images (Ruifrok and Johnston, 2001) to separate the image into a clonal mark channel and a nuclear channel. Using the nuclear channel it uses morphology operations to identify a number of candidate crypts and then applies a model based classification step to select the true crypts (Figures S1A and S1B). The algorithm has been constructed so as to be robust to the variability in staining intensities and crypt morphologies observed within and between slides, and while dependent on the quality of the slide, typically identifies ~95% of crypts and makes around ~5% false positives. It was programmed in python using opencv (Bradski, 2000), openslide (Goode et al., 2013) and Vips (Martinez, 2005) as its image processing libraries.

Pipeline and quality control

Single cell clones are often small and faint, which makes it hard for the algorithm to distinguish them from small artifacts from the staining process. In order to improve the quality of the data we included all detected clones regardless of size and stain intensity and introduced a manual quality control stage. The algorithm was altered to produce an image list of candidate clones ordered by stain intensity and clone size, along with a filled-in spread sheet for manual QC adjustments.

All the outputs were set up to be visualized from a web-browser (Figure S1). Every analyzed slide had associated to it a fully annotated slide image that could be visualized using Google maps (https://github.com/evildmp/VirtualMicroscope) (Figure S1D), a Google-docs spread sheet with the detected clones (Figure S1E) and a web-based image list with the detected clones (Figure S1F). In order to manage the collections of ~1,000 slides we used Zegami [https://zegami.com/] https://zegami2016.molbiol.ox.ac.uk/crypt_1 (Figure S1G).

Tissue block viewer

The estimation of the mutation rate requires scoring transit amplifying (TA) clones. To find TA clones crypts were tracked in 3D. Serial sections of a tissue block were analyzed individually as described above. Tissue sections can rotate as they are placed on the slide, which means that images from serial sections do not always align. We developed a Block Viewer tool that takes all slides from the same block, aligns them and highlights the QCed clones. The viewer shows zoomed out images of two tissue sections next to each other with the detected clones highlighted. A slider allows moving through the block sections. The sections are clickable showing zoomed in versions of the clicked region for both tissue sections, allowing the same crypt to be visualized in high resolution through the block (Figure S2A).

The tool works by first taking a heavily down-sampled version of the image and applying opencv's orb method to detect key points. The key points are then used to find the rotation and translation required to align the images via RANSAC fitting. When clicking on the zoomed out and rotated version of the image we undo the transformation, extract the correct area of the image and transform again for the zoomed in coordinate system. We found that 80%–90% of images could be aligned this way. In general, the sections that failed were cases where the sections were very far apart and therefore looked very different.

Estimating the mutation rate for the mPAS clonal mark

As described in Kozar et al., (2013) it is possible to infer the mutation rate using clones arising in the TA compartment. The estimation is very simple and requires calculating the ratio of TA clones scored in tissue section over the total number of cells scored. Using the clones found from the algorithm with the block viewer we scored the TA clones. Marked mPAS$^+$ cells were compared across matched sections. Each mPAS$^+$ clone was manually scored to identify and record those cases where the mPAS positivity was

not part of a larger pre-existing clone. In order to calculate the total number of goblet cells informative for mPAS, Alcian blue staining was performed, this enabled the average number of goblet cells per crypt area to be calculated and was used to provide cellular values when calculating the mutation rate (Figure S2). To estimate the number of goblet cells scored, we used the area of the crypts in these sections to estimate the goblet cells for each crypt. To be able to map the area of the crypts to the number of goblet cells we generated a separate dataset where we manually scored goblet cells stained by Alcian blue as well as the corresponding crypt area for 274 crypts of a range of sizes and from 14 different tissue slides (Figures S2B–S2D). Using a non-linear spline regression, we used this dataset to derive a mapping from crypt area to number of goblet cells.

Evaluation of X-linked genes for clonal analysis

Genes encoded on the X chromosome and subject to X-inactivation were evaluated as potential clonal marks with 111 genes of which the encoded protein gave strong epithelial staining according to the Human Protein Atlas (HPA) annotations. Of these 20 were selected as showing consistent staining intensities across cell types and throughout the epithelium. Eight of them were screened as potential clonal marks by IHC staining of large area sections of at least 25 aged individuals (> 70 years) and a minimum of 70K crypts (Table S2).

Immunohistochemistry

Sections of 5 um were cut from formalin-fixed paraffin-embedded samples onto charged slides. Sections were de-waxed and re-hydrated followed by heat-induced epitope-retrieval using 10 mM Tri-sodium Citrate buffer pH6.0. Sections were blocked in 3% H_2O_2 in methanol and subsequently blocked in 10% Donkey Serum for 30 minutes. Slides were then incubated with anti-MAOA or anti-STAG2 antibodies (MAOA: mouse monoclonal, Santa Cruz Biotechnology and Rabbit polyclonal, Sigma, STAG2: goat polyclonal, LifeSpan BioSciences and Rabbit polyclonal, Sigma) overnight at 4°C. Sections are incubated with biotin-SP-conjugated AffiniPure donkey anti-mouse or anti-goat, Jackson ImmunoResearch, both 1:500 in PBS-T) for 40 minutes at room temp followed by incubation with Vectastain® Elite® ABC reagent (Vector Laboratories) for 40 minutes. This was followed by immunoperoxidase detection using a liquid DAB + substrate chromogen system (Dako). Sections were then counterstained in hematoxylin before dehydration, clearing and mounting.

Targeted amplicon KRAS sequencing

Genomic DNA was extracted from FFPE sections using a QIAamp DNA FFPE tissue kit (QIAGEN-56404) according to manufacturer's instructions. gDNA template was PCR amplified in duplicate for each sample (NEB Phusion DNA polymerase, HF buffer, 2 mM $MgCl_2$, 200 μM each primer, 500 nM dNTPs). Forward and reverse gene specific primers fused with Fluidigm Corporation barcoding CS1 and CS2 adaptor sequences (forward - ACACTGACGACATGGTTCTACA-GGTGGAGTATTTGATAGTGTATTAACC and reverse - TACG GTAGCAGAGACTTGGTCT-TAGCTGTATCGTCAAGGCAC) were used. The resulting amplicon comprised 159bp of KRAS sequence encompassing codons 12 and 13. Amplicons were diluted and re-amplified with Fluidigm barcoding primers (incorporating a unique sample barcode and Illumina P5 and P7 adaptor sequences), pooled and subjected to 150 bp paired end sequencing on an Illumina MiSeq platform.

QUANTIFICATION AND STATISTICAL ANALYSIS

Statistical Analysis of clone data

A general note on simulations and the mathematical model

Throughout the manuscript we have made use of a mathematical model (described below) that models the acquisition of a mutation, the competition of the mutant stem cell with the other stem cells and, once fixed, the fission of the mutant crypt.

Additionally we challenge the model with two more complex scenarios to study whether more complexity is warranted. We do this using simulations that encode the same assumptions as the mathematical model but with additional behaviors. Specifically, we check the effect of fusion on patch size and the effect of double hits on the clonal dynamics.

The implementations of these two simulations are very different as, in order to simulate fusion, one has to simulate the spatial dynamics of the clone and surrounding crypts (a monoclonal crypt can fuse with another monoclonal crypt or to an unlabelled crypt leading to a partial), whereas the double hit simulations require just one crypt to be modeled but require tracking the individual cells and how many mutations each one has.

All the simulations were coded in python using the numba library for speed.

Statistical inference

All data fitting was done using the statistical models described in the fitting sections below and sampled from using Rstan (Carpenter et al., 2017). Rstan was run using 5 chains of 10,000 iterations and a thinning of 5. The default parameters were used for the sampler, though where necessary, the models were reparamertised and run parameters adapted. Convergence was checked using the scale reduction factor provided by Rstan.

Within the main text estimates are presented as credible intervals (CI) or alternatively as a margin of error (ME) expressed as a median and 1.96 times the standard deviation of the posterior. For cases where new parameters, are calculated that are functions of the inferred parameters we apply the function to all the posterior mcmc samples and present the median and 1.96 times the standard deviation of the transformed samples.

For some of the cases below, Gaussians were used to model the population variability of a parameter defined in the [0, 1] range, for these cases the range of the parameter was specified in Stan.

Statistical model for TA clones

Patients were selected based on tissue block size that so as to be able to estimate a mutation rate per block. In some cases we had several such blocks for the same patient, which we used within the statistical model to estimate the within patient variability and experimental error. A hierarchical model was used as follows, assuming we measure $k_{i,b}$ TA clones for patient i in block b, the number of goblet cells measured is $G_{i,b}$ and the mutation rate for patient i is α_i the counts are distributed as

$$q_{i,b} \sim Normal(\alpha_i, \sigma_{error})$$

$$k_{i,b} \sim Binomial(G_{i,b}, q_{i,b})$$

We calculate the distribution of the mutation rate in the patient population as

$$\alpha_i \sim Normal(\mu_\alpha, \sigma_\alpha)$$

The priors used were:

$$\mu_\alpha \sim Beta(1/2, 1/2)$$
$$\sigma_\alpha \sim Beta(1/2, 1/2)$$
$$\sigma_{error} \sim Beta(1/2, 1/2)$$

Continuous labeling of a neutral mutation

Here we describe the continuous labeling model that can be found in Kozar et al., (2013). It has been shown that crypts are maintained by an equipotent population of stem cells at the crypt base that constantly replace each other in a stochastic fashion (Lopez-Garcia et al., 2010; Snippert et al., 2010). The equations that govern the change in clone size with time assume we start tracking the progeny of a clone of size 1 stem cell at t = 0. The probability of a crypt having clone of size n (for 0 < n < N) at time t is:

$$P_n(t) = \frac{2}{N}\sum_{m=1}^{N-1} \sin\left(\frac{\pi m}{N}\right)\sin\left(\frac{\pi m n}{N}\right) e^{-4\lambda \sin^2\left(\frac{\pi m}{N}\right)t}$$

Here n is the number of stem cells that make up the clone, N is the total number of stem cells in the crypt base and λ is the rate of stem cell replacement. For the probability of the clone being of maximum size, i.e., a monoclonal crypt:

$$P_N(t) = \frac{2}{N}\sum_{m=1}^{N-1} (-1)^{m+1} \cos^2\left(\frac{\pi m}{2N}\right)\left(1 - e^{-4\lambda \sin^2\left(\frac{\pi m}{2N}\right)t}\right)$$

For our case if we are tracking mutationally tagged clones. If we take the mutation rate to be α the rate at which a crypt will get a mutationally activated clone will be

$$\kappa = \alpha\lambda N$$

If we write down the stochastic master equation for this:

$$\frac{dQ_0}{dt} = -\kappa Q_0$$

$$\frac{dQ_1}{dt} = \kappa Q_0$$

We can solve and get

$$Q_1(t) = \left(1 - e^{-\kappa t}\right)$$

As the mutation rate is very low we can use a Taylor expansion to get

$$Q_1(t) \approx \kappa t$$

New clones of size one stem cell are appearing continuously over time, assuming the mutation has no effect on the stem cell dynamics, the clone size will evolve according to the equations above. To model the probability of clone size over time we can use the integral

$$C_n(t) = \int_0^t \frac{dQ_1}{d\tau}(\tau)P_n(t-\tau)d\tau$$

Which assumes that the clones that disappear due to stem cell competition have a negligible effect on Q_0.

Solving for the non-monoclonal clones and pooling them to get the partial clone prediction we get:

$$C_{partial} = \alpha\frac{N(N-1)}{2} - \frac{\alpha}{2}\sum_{n,m=1}^{N-1}\frac{\sin\left(\frac{\pi m}{N}\right)\sin\left(\frac{\pi mn}{N}\right)}{\sin^2\left(\frac{\pi m}{2N}\right)}e^{-4\lambda \sin^2\left(\frac{\pi m}{2N}\right)t}$$

For the monoclonal clones we get

$$C_{monoclonal} = \alpha\lambda t - \frac{\alpha}{2}\sum_{m=1}^{N-1}\frac{(-1)^{m+1}}{\tan^2\left(\frac{\pi m}{2N}\right)}\left(1 - e^{-4\lambda \sin^2\left(\frac{\pi m}{2N}\right)t}\right)$$

The effect of the exponential term is quickly lost, leading to a constant term for the partials and a linear function for the monoclonals.

Continuous labeling of a non-neutral mutation

Vermeulen et al., (2013) showed that certain mutations can affect the clonal dynamics. Furthermore they showed that these altered dynamics could be parameterized by introducing a replacement probability, P_R. The equations for the non-monoclonal and monoclonal clones are as follows:

$$R_n(t) = \frac{2}{N}\left(\frac{\beta}{\gamma}\right)^{\frac{1}{2}(n-1)}\sum_{m=1}^{N-1}k_{m,n}e^{-h_m t}$$

$$R_N(t) = \frac{2\beta}{N}\left(\frac{\beta}{\gamma}\right)^{\frac{1}{2}(N-2)}\sum_{m=1}^{N-1}\frac{k_{m,N-1}}{h_m}\left(1 - e^{-h_m t}\right)$$

Where the following shorthand has been used:

$$\gamma = 2\lambda(1 - P_R)$$
$$\beta = 2\lambda P_R$$
$$k_{m,n} = \sin\left(\frac{\pi m}{N}\right)\sin\left(\frac{\pi mn}{N}\right)$$
$$h_m = 4\sqrt{\gamma\beta \sin^2\left(\frac{\pi m}{N}\right)} + \gamma + \beta + \ - 2\sqrt{\gamma\beta}$$

While the drift dynamics are different to the neutral case, the dynamics of the appearance of the initial mutations are the same; therefore we can derive the continuous labeling equations in the same way

$$\widehat{C}_n(t) = \int_0^t \frac{dQ_1}{d\tau}(\tau)R_n(t-\tau)d\tau$$

Which leads to

$$\widehat{C}_{partial}(t) = \frac{2\kappa}{N}\sum_{m,n=1}^{N-1}\left(\frac{\beta}{\gamma}\right)^{\frac{1}{2}(n-1)}\frac{k_{m,n}}{h_m}\left(1 - e^{-h_m t}\right)$$

$$\widehat{C}_{monoclonal}(t) = \frac{2\beta\kappa}{N}\left(\frac{\beta}{\gamma}\right)^{\frac{1}{2}(N-2)}\sum_{m=1}^{N-1}\frac{k_{m,N-1}}{h_m}\left(t - \frac{1}{h_m}\left(1 - e^{-h_m t}\right)\right)$$

For the sake of brevity we do not expand the equations, however it is worth noting that much like the neutral mutations, after a short initial period the monoclonals follow a linear equation and the partials converge to a constant value. For both the neutral and non-neutral cases the equations are proportional to the mutation rate, meaning that the ratio of the slope of the monoclonal accumulation over the partials gives a value that is independent of the mutation rate. This can be used as a way of comparing the clonal dynamics for different mutations.

Fitting the monoclonal clones and partial clones

As the probability of a crypt containing a monoclonal clone at time t is a linear function we fit the following model to the monoclonal data:

$$p_i = a_i(t_i - t_0)$$
$$k_i^{mono} \sim Binomial(C_i, p_i)$$

Where k_i^{mono} is the number of monoclonal crypts found for patient i, C_i is the number of crypts in the tissue sample, t_i is the patient age, a_i is the slope of the monoclonal accumulation for patient i and t_0 is the x axis intercept. As we expect the mutation rate to have some variation between individuals, as well as the drift parameters, we allow each patient to have its own slope, using a hierarchical model

$$a_i \sim Normal(\mu_a, \sigma_a)$$

The priors on the parameters are as follows

$$\mu_a \sim Gamma\left(10^{-2}, 10^{-2}\right)$$
$$\sigma_a \sim Gamma\left(10^{-2}, 10^{-2}\right)$$
$$t_0 \sim Normal(0, 10)$$

Note how we are allowing t_0 to be negative. While the stem cell dynamics equations suggest that the y-intercept should be negative, and as such the x-intercept should be positive it is possible that clones might arise during development that would increment the y-intercept allowing for the x-intercept to become negative. We choose a value that encompasses ~20 years to either side of the origin to allow a wide range of values, however restricting implausible values.

We follow a similar analysis for the partial clones.

$$k_i^{partial} \sim Binomial(C_i, b_i)$$
$$b_i \sim Normal(\mu_a, \sigma_b)$$

With priors

$$\mu_b \sim Gamma\left(10^{-2}, 10^{-2}\right)$$
$$\sigma_b \sim Gamma\left(10^{-2}, 10^{-2}\right)$$

Effect of crypt fusion on patch size

A recent study has shown that crypts not only undergo fission, where a crypt divides into two crypts, but they can also fuse with a neighboring crypt thus combining the stem cell pools. The study found that fission and fusion are balanced, both occurring at the same rate.

At the clonal level fusion can cause a mutant crypt to join with a non mutant producing a partially mutant crypt or two mutant crypts can join forming a single mutant crypt. This introduces a spatial aspect to the model, which complicates an analytical approach. To assess the effect of fusion we implement a stochastic simulation algorithm which uses the gillespie algorithm. The simulation models a field of crypts and implements the mutation process, stem cell drift, fission and fusion, including the spatial aspects as well as the two types of fusion events described above.

The simulations showed that relative patch size is dominated by fission, with fusion having a very modest effect (Figure S4).

Crypt fission and mutation burden

We model crypt fission as a Yule-Furry pure birth process. The general solution to this process is:

$$\widehat{F}_n(t) = \binom{n-1}{n-n_0} e^{-\rho n_0 t}\left(1 - e^{-\rho n_0 t}\right)^{n-n_0}$$

Where n_0 is the patch size at time t = 0 and ρ is the rate of crypt fission. In order to calculate the patch size distribution over time given that the monoclonal crypts appear following a known function we can use a similar calculation as for the continuous labeling equations. We fix $n_0 = 1$ and integrate:

$$F_n(t) = \int_0^t \frac{dC_{monoclonal}}{d\tau}(\tau)\widehat{F}_n(t-\tau)d\tau$$

Ignoring the exponential term from $C_{monoclonal}$ which has a negligible effect, we find

$$F_n(t) = \Delta C_{monoclonal}\frac{\left(1 - e^{-\rho t}\right)^n}{\rho n}$$

Here $\Delta C_{monoclonal}$ is the slope of the monoclonal accumulation. This equation also holds for mutations that affect clonal drift. We use this equation to estimate the mutant burden per million crypts used in the main text:

$$B(t) = 10^6 \sum_{n=1}^{\infty} nF_n(t)$$

Relative expansion coefficient

In order to derive a metric for each mutation that allows comparison of the ability of the mutation to spread through the tissue we calculate the burden of a mutation averaged over the lifetime of the individual. We then calculate the ratio of average burden between a given mutation and the wild-type parameters. By fixing the mutation rate to the same value for both average burden estimates, the mutation rate disappears from the ratio.

$$I^{mutant} = \frac{\frac{1}{100}\int_0^{100} B^{mutant}(t)dt}{\frac{1}{100}\int_0^{100} B^{WT}(t)dt}$$

We refer to this value as a relative expansion coefficient (C_{exp}). The values used in the main text were calculated numerically using the burden equation described in the previous section.

Statistical model for patch sizes

The patch size equation depends on the slope of the monoclonals, which we can infer from the monoclonal data. However in order to minimize the uncertainty in the crypt fission estimation, we calculate the equation for the relative distribution of patch sizes that does not depend on the slope of the monoclonals:

$$f_n(t) = \frac{F_n(t)}{C_{monoclonal}(t)} = \frac{(1 - e^{-\rho t})^n}{\rho n t}$$

This is the same equation used by Baker et al., (2014). We also apply a correction for the confounding effect of two unrelated clones randomly being found next to each other and counted as a patch. If a tissue sample has k clones, C crypts and each crypt has δ neighboring crypts the proportion of clones that form random doublets will be:

$$D = \sum_{i=1}^{k-1} \frac{\delta}{k} \frac{i}{C - i} \approx \delta \frac{k-1}{2C}$$

We do not calculate the probability of patches larger than two appearing due to chance as the probability of these events will be negligible. When fitting the model to the data we add D to f_2 and subtract D from f_1.

As a first step for the fitting we filter samples with no clones as we are fitting the relative patch size. Again we use a hierarchical model to account for patient-to-patient variability. If g_i is a vector of measured patch sizes, t_i is the age of the patient, ρ_i is the fission rate for that patient we have

$$g_i \sim Multinomial(f(\rho_i, t_i))$$
$$\rho_i \sim Normal(\mu_\rho, \sigma_\rho)$$

Where f is the vector of probabilities of each patch size calculated from the fission equation and corrected as specified above. The priors used for the population parameters are

$$\mu_\rho \sim Gamma(10^{-2}, 10^{-2})$$
$$\sigma_\rho \sim Gamma(10^{-2}, 10^{-2})$$

Sequential mutations

The mutation of *STAG2*, a gene that when mutated is associated with chromosomal instability, was found to have a biased behavior. The fact that *STAG2* is associated to chromosomal instability raises the question of whether the biased behavior is the consequence of further unmeasured mutations enabled by the chromosomal instability or directly caused by *STAG2*. To find which might be the most likely scenario we run simulations where we assume that a first neutral mutation raises the mutation rate of a second mutation that biases drift.

The simulation uses the Gillespie Algorithm to simulate a single crypt with N stem cells, each of which starts with no mutations and can acquire a first mutation which doesn't change the drift dynamics, however the mutant cells now have an enhanced probability of a second mutation which does lead to a bias. The simulation produces two outputs, the monoclonal and partial crypts for the first mutation, regardless of whether or not they have the second mutation (this would be what we measure with *STAG2*) and also outputs the full and partial crypts with both mutations (as you can't have mutation 2 without 1).

If we can only measure mutation 1, as happens with *STAG2*, in order to see altered dynamics caused by mutation 2 the mutation has to occur while mutation 1 has not yet become monoclonal, otherwise we would measure no difference (Figures S3H and S3I).

Analysis of KRAS sequencing data

Analysis of raw data

Corresponding forward and reverse reads were combined into a single consensus sequence using PANDAseq 2.11 with default options (Masella et al. 2012). Amplicon sequences were removed if they did not begin and end with the forward and reverse gene specific primer sequences respectively and/or were incorrect overall length (> 164 bp). Both read number ($\geq$1000) and FFPE section quality ($\geq$1000 crypts identified in a serial section) were used to filter data resulting in 126 patients being processed for further analysis. The frequency of all four nucleotides at all amplicon positions was calculated for each sample using a custom PERL script (NUCLEOTIDE_COUNT_FOR_HASH.pl). The resulting flat file was processed by HASH_3.pl to calculate the percentage frequency for every position/nucleotide for each sample and then the mean frequency and st.dev. of all samples, on a given sequencing run, for each particular position/nucleotide. Mutations were called if a variant nucleotide exceeded either; 4x the mean allele frequency or the mean allele frequency + 3.209 st.dev., and there were a minimum of 10 variant reads (the mean read depth per sample was 10535 [±6002 st.dev.]). Both replicates of a sample had to be called with the same mutation for the sample to be considered mutated. The actual MAF for subsequent use was calculated by subtracting the mean allele frequency for that position/nucleotide.

Statistical model for patch size estimation

From this analysis sections from 13 of 126 patients had detectable MAF in range of 0.2%–1.8% with an estimated sensitivity of detection of 10^{-3} (Table S3). To analyze this allele frequency data we first convert it to mutation burden. To do so we note that if in a section

of tissue we have m mutant crypts, C total crypts and n cells per crypt the ratio of mutant copies of a gene to total copies of the gene will be

$$f_{allele} = \frac{mn}{2Cn}$$

which means that the allele frequency is half of the mutation burden.

In order to model this data we can use the equation for patch sizes derived earlier, namely

$$F_n(t) = \Delta C_{monoclonal}\frac{\left(1 - e^{-\rho t}\right)^n}{\rho n}$$

Which gives us the probability of finding a patch of size n at age t. The model has two parameters the fission rate ρ and the monoclonal accumulation rate ΔC_{fix}. These are the two parameters we wish to infer from the data.

The statistical fitting must account for the fact that there is a detection limit below which there may be clones but we cannot detect them. This threshold is very different for the two data types we are fitting. We set up the statistical model so that if the mathematical model predicts that there should be a patch but we measure none, as long as it is below the specified detection threshold, it does not penalise the fit.

We first take the measured allele frequency and convert them to mutation burden, we then use the number of crypts from that sample to convert the burden into patch size. For the amplicon sequencing we know how many crypts we have in the sample from the image processing. For the ACB-PCR we know that the amount of DNA used is 300,000 copies so we estimate the number of crypts to be 150,000.

We cannot directly use the patch size equation as we need to accommodate the fact that we have a range of possible patch sizes of which each patient will only have one, also the probability of not detecting a patch will need to be calculated depending on the values of the parameters.

We model each patient sample as a multinomial with three categories, probability that a crypt has no detectable clone q_0, probability q_1 that we see a patch of size n (where n is the observed patch size) and q_2 the probability of all the remaining patch sizes, used to normalize the multinomial $q_2 = 1 - (q_0 + q_1)$. We calculate q_0, which incorporates the detection threshold as

$$q_0 = \sum_{n=0}^{n_{limit}} F_n(t)$$

Here n_{limit} is the largest patch size that would not be detected. We calculate the probability of no clone with

$$F_0 = 1 - \Delta C_{monoclonal} t$$

The likelihood will be

$$n_i \sim Mutinomial(q)$$

Where q is the vector described above and n_i is a vector of 3 counts for patient i: total crypts, zero or one if there is a patch and 0 for the third category.

The priors used for the two parameters are

$$\rho \sim half\text{-}normal(0, 0.5)$$
$$\Delta C_{fix} \sim half\text{-}normal\left(0, 10^{-4}\right)$$

For the results of the two datasets to be comparable we need to scale ΔC_{fix} by the number of mutations we look at. In the case of ACB-PCR we just look at one, whereas with the targeted sequencing we look at 12 possible mutations.

DATA AND SOFTWARE AVAILABILITY

Please refer to the URLs for the following: collection of slides stained with mPAS, https://zegami2016.molbiol.ox.ac.uk/crypt_1; Google maps pathology viewer, https://iime.github.io/virtualmicroscope/; image segmentation software DeCryptICS, https://github.com/MorrisseyLab/DeCryptICS; crypt stochastic drift software CryptDriftR, https://github.com/MorrisseyLab/CryptDriftR; *KRAS* sequencing data, NCBI Study Accession SRP139051; and sequence analysis scripts, https://github.com/kemp05/KRAS-sequencing/blob/bd759fc005f89198116a8be97531bd9ec5f5f5ca/NUCLEOTIDE_COUNT_FOR_HASH.pl and https://github.com/kemp05/KRAS-sequencing/blob/bd759fc005f89198116a8be97531bd9ec5f5f5ca/HASH_3.pl.

Cell Stem Cell, Volume *22*

Supplemental Information

Fixation and Spread of Somatic Mutations in Adult Human Colonic Epithelium

Anna M. Nicholson, Cora Olpe, Alice Hoyle, Ann-Sofie Thorsen, Teja Rus, Mathilde Colombé, Roxanne Brunton-Sim, Richard Kemp, Kate Marks, Phil Quirke, Shalini Malhotra, Rogier ten Hoopen, Ashraf Ibrahim, Cecilia Lindskog, Meagan B. Myers, Barbara Parsons, Simon Tavaré, Mark Wilkinson, Edward Morrissey, and Douglas J. Winton

Figure S1. Image analysis pipelines. Related to Figure 1 and STAR methods

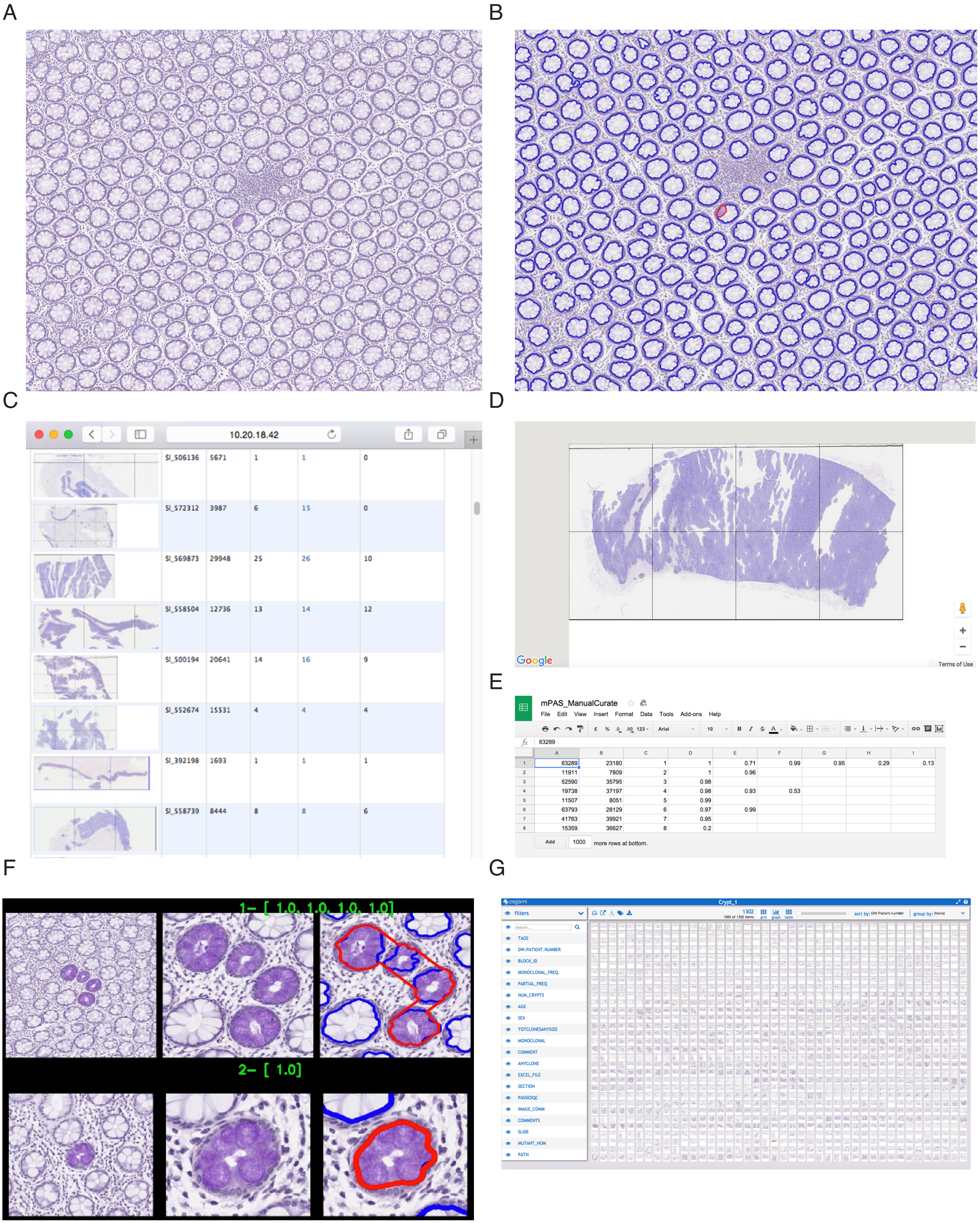

Figure S2. Determining a de novo mutation rate in human colon.
Related to Figure 1 and STAR methods

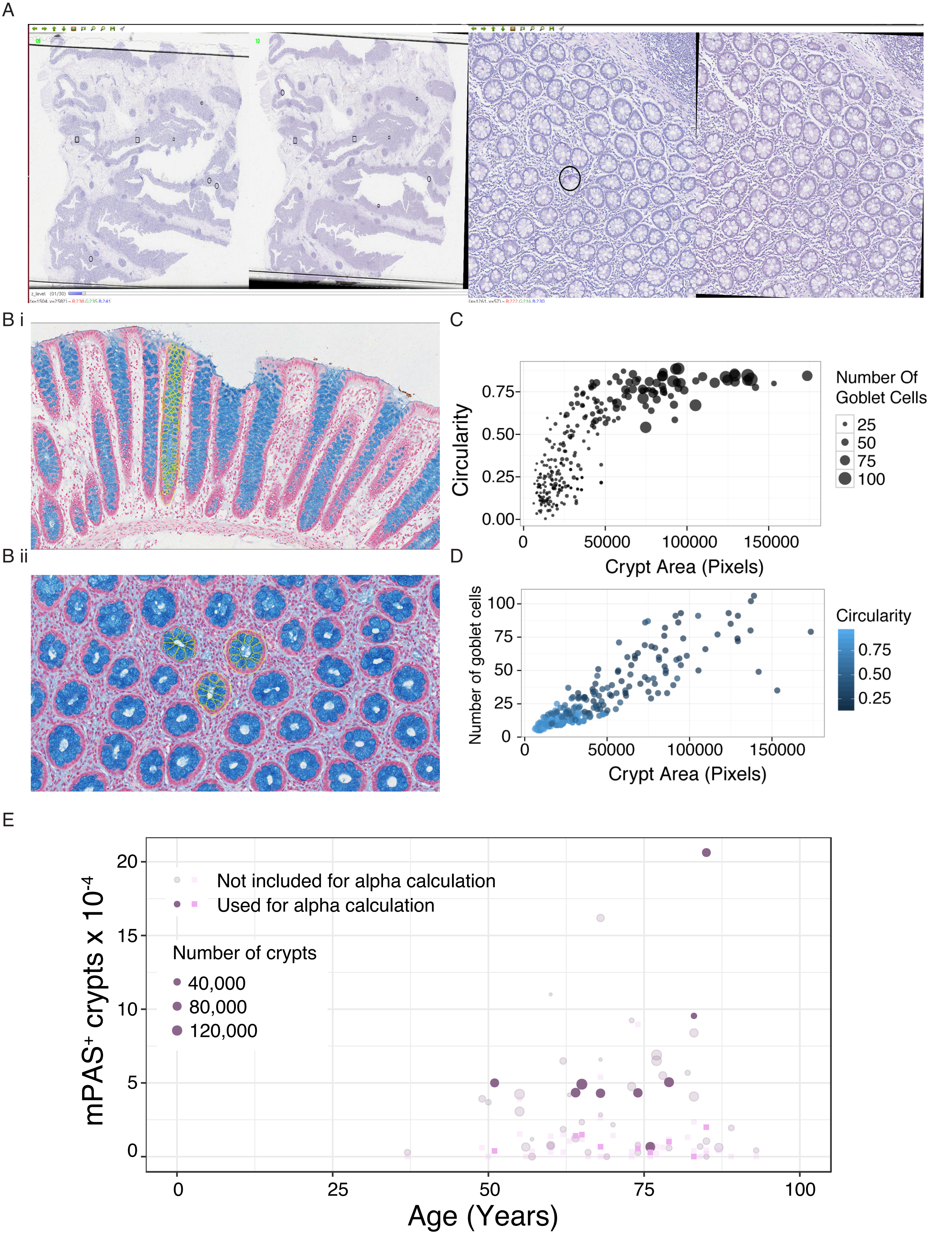

Figure S3. Split MAOA data set, conversion rates and sequential mutation simulations, Related to Figure 2, Figure 3 and STAR Methods

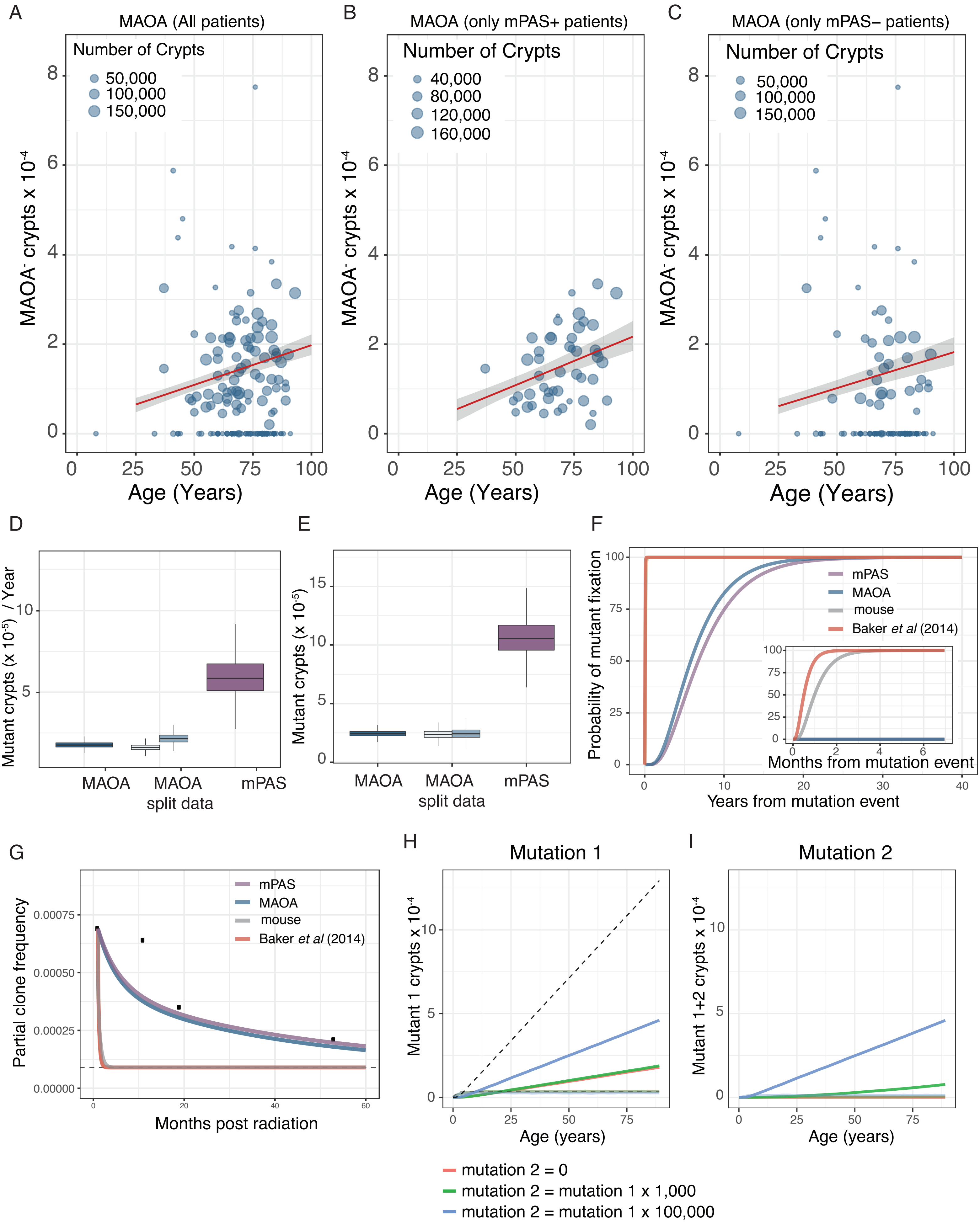

Figure S4. Effects of fission and fusion on patch size in a WT and oncogenic setting, Related to Figure 4 and STAR Methods.

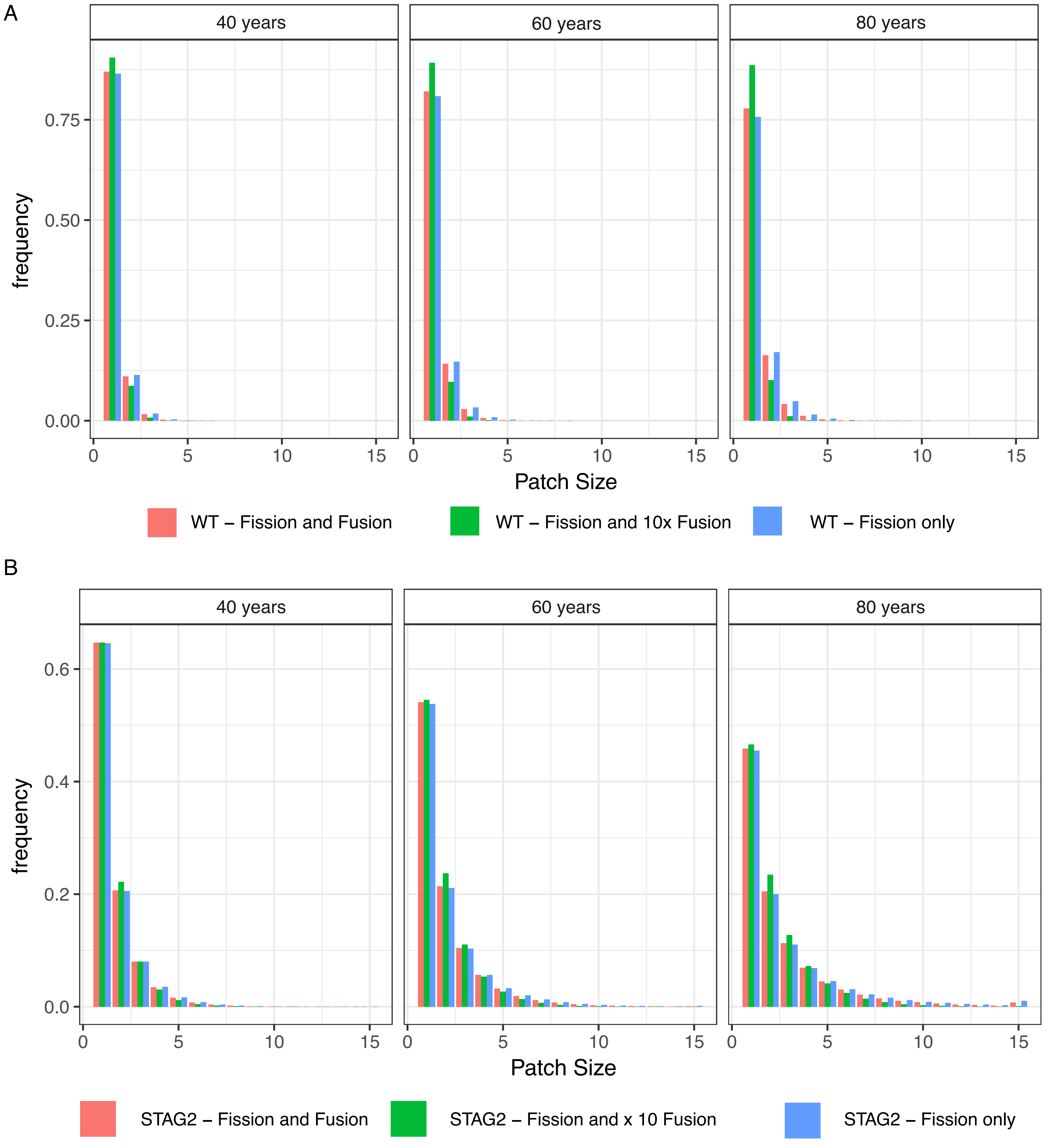

Supplemental Figure Legends

Figure S1. Image analysis pipelines. Related to Figure 1 and STAR Methods

(A, B) mPAS stained sections are scanned and images processed to identify crypts and candidate clones within the section. Before processing (A). After processing (B) crypts are outlined (blue) and mPAS positive clones identified (a PPC in this case) (red) (C) View of web-browser populated with tiles for each mPAS stained section, a linked image ID, calculated crypt number and number of detected clones. (D) An example of the use of Google maps to visualise images. Each processed mPAS stained section can be opened using Google maps via the link in the web-browser to visualise detected clones within the tissue section. (E) A Google sheet is produced for each stained and processed section with information on all clones detected, including co-ordinates and number of crypts within a patch. Using this information each clone is manually validated. (F) An image list of candidate clones are viewed from a link in the web-browser, each clone is numbered and a low power and high power pre-processed image of the clone is shown alongside the processed image. Within the list, images are ordered by stain intensity and size. (G) View of the Zegami software, Zegami was used to aid in visualisation of the many hundreds of slides included in this study. Alongside the images, complete data including patient information such as age and sex as well as number of clones can be observed within the software.

Figure S2. Determining a de novo mutation rate in human colon. Related to Figure 1 and STAR Methods

(A) Rotated serial sections can be viewed side by side on the screen. The detected clones are highlighted on each section, and a slider seen on the bottom of the screen enables movement through the serial sections. Clicking on an area of the low power section brings up a high power image of that area on both serial sections in view, this enables the same crypt to be visualised throughout the block. (B) Sections from human colon stained using Alcian blue/Periodic Acid Schiff's. Images from over 200 crypts were annotated using Fiji (ImageJ) to determine the crypt area and the number of goblet cells within each crypt scored (yellow lines). Crypts from both *en edge* (i) and *en face* (ii) sections were included in the analysis. (C) Dot plot shows roundness (0-1) against crypt area in pixels, where the size of the dot is relative to the number of goblet cells within the crypt. (D) Dot plot shows the number of goblet cells against crypt area in pixels, where the more circular the crypt the darker blue the point. Data from manual scoring shows larger crypts contain more goblet cells, and that rounder crypts are generally smaller. (E) For each patient the frequencies of $mPAS^{+}$ WPC (circles) and PPC (squares) are plotted against patient age (years), in this plot the individuals used to calculate the mutation rate are highlighted in darker points compared to those not used for the calculation.

Figure S3. Split MAOA data set, conversion rates and sequential mutation simulations. Related to Figure 2, Figure 3 and STAR Methods

(A-C) Frequency plots of WPC for MAOA deficient clones with regression analyses showing$D\Delta C_{fix}$ for MAOA plotted in red with 95% CI shaded in grey. All 152 patients are included in (A), while the 48 mPAS+ patients are only plotted in (B) and the 104 mPAS- patients are plotted in (C). (D) Box plot showing the ΔC_{fix} found for

each patient set including the credible intervals, and (E) box plot showing the fitted frequency of partially populated crypts along with their credible intervals in each patient set. (F-G) Comparison of monoclonal conversion rates. Mutant fixation times by crypt monoclonal conversion plotted using parameters derived from: (F) spontaneous mPAS and MAOA clone data and those stated in Baker *et al* (2014) and for mouse colonic epithelium (Vermeulen *et al*, (2013)). (G) Model predictions of monoclonal conversion times using published human "pulse chase" data for mPAS PPC clones following irradiation. Points are data from Campbell *et al* 1996. Lines show model inference predictions. (H-I) Results from simulations of sequential mutations. The graphs show the full and partial clone frequency for simulations of double mutations. (G) The dotted lines show the observed data for STAG2. The coloured lines show the predicted clone frequencies for a comparable gene (Mutation 1) assuming it is neutral with respect to stem cell replacement but conferring an altered mutation probability at the rates shown on a second locus (Mutation 2) that confers an advantage on replacement. (I) Shows the predicted frequencies of clones containing both Mutation 1 and Mutation 2.

Figure S4. Effects of fission and fusion on patch size in a WT and oncogenic setting. Related to Figure 4 and STAR Methods

Results of simulations for different fusion rates showing that relative patch size is dominated by the fission rate, with the fusion rate having to be 10 times higher than fission to see an appreciable effect. (A) Using mPAS inferred parameters and varying the fusion rate. The fusion rates used are 0, balanced fusion and fission rates and fusion rate x 10. (B) Using Stag2 inferred parameters with the fusion rates as described for A.

Table S1. Summary of patients included in the study, Related to STAR Methods

Characteristic		Set (n = 187)
Age	Minimum	8
	Maximum	93
	Median	69
Sex	Male	97
	Female	89
Colonic Region	Left	100
	Right	66
	Unknown	21

Table S2 – Summary of X-chromosome encoded proteins investigated as part of this study. Related to STAR Methods

Antigen	Antibody	Conc.	Crypts screened	Comments	DNA repair/pro-oncogenic associated
Apex2	HPA030872	0.5ug/ml	73,529	No clones detected	Yes
CXorf57	HPA001374	0.5ug/ml	125,150	No clones detected	No
GPKOW	HPA001894	4ug/ml	83,567	No clones detected	No
MAOA	SC-271123<br>HPA059299	1ug/ml<br>1ng/ul	6,815,804	1,052 clones detected<br>Validation	No
POLA1	HPA002947	0.1mg/ml	168,395	No clones detected	Yes
RbAp48/RbAp46	R3779	1.2ug/ml	121,377	No clones detected	Yes
Stag2	LS-B11284<br>HPA002857	1ug/ml<br>2.1ug/ml	5,402,413	8,743 clones detected<br>Validation	Yes
THOC2	HPA047921	0.1mg/ml	178,854	No clones detected	No

Table S3 – Data from normal colonic mucosa of 20 patients showing *KRAS*G12D mutant allele frequency (Log10). Related to Figure 4 and STAR Methods

Sample	Age	Log10 *KRAS* codon 12 G12D mutant frequency (GAT)
1	30	-4.61
2	31	-4.54
3	33	-4.58
4	34	-4.68
5	46	-4.19
6	48	-5.14
7	49	-4.87
8	49	-5.19
9	50	-3.78
10	50	-4.57
11	52	-4.57
12	53	-4.59
13	54	-3.32
14	56	-2.83
15	58	-3.94
16	72	-3.82
17	79	-4.72
18	79	-3.85
19	88	-3.25
20	94	-4.47
